# A multi-dimensional CNN-Bi-GRU for IoT-based brain–computer interface in early epileptic seizure detection

**DOI:** 10.1093/biomethods/bpag010

**Published:** 2026-02-17

**Authors:** Biplov Paneru

**Affiliations:** Independent Researcher, Kapan 3, Shivashaktinagar, Kathmandu, 44600, Nepal

**Keywords:** epileptic seizure, explainable AI, SHAP analysis, EEGNet, MDCBG, Internet of Medical Things (IoMT)

## Abstract

The study focuses on seizure detection using EEG data from Mendeley. An early-alert IoT-BCI system is designed to simulate real-time support for patients during seizures. The proposed Multi-Dimensional CNN-Bi-GRU (MDCBG) outperforms hybrid deep learning models, achieving 97.43% accuracy, surpassing baseline EEGNet (92.17%) and CTNET (85.11%), along with models evaluated through ablation studies on seizure vs. non-seizure prediction. The proposed model, along with other models like Bi-GRU with attention, Bi-LSTM-GRU, and XGBoost, also performs well on classifying various types of seizures. SHAP analysis shows Channel 5 contributes most to predictions. An IoT-based automation system is simulated on seizure detection for triggering micro devices near the patient’s environment. This approach supports early seizure warning and guides home-automation strategies to assist patients.

## Introduction

As machine learning (ML) and artificial intelligence (AI) continue to advance, there is an increasing emphasis on using these technologies to improve clinical applications. Early disease diagnosis and prediction to facilitate early preventive intervention is a major objective in healthcare [1]. Researchers have focused a lot of emphasis on epileptic seizures, which are neurological disorders marked by particular patterns in electroencephalography (EEG) recordings. EEG signal feature extraction and pattern classification have been successfully accomplished using machine learning (ML) and deep learning (DL) approaches [2]. Recurrent seizures that result in unconsciousness or muscle spasms are a common neurological disorder called epilepsy, which has a major negative influence on a patient’s health, everyday activities, and general well-being [3].

Because EEG recordings offer vital information about the electrical activity of the brain, they are widely employed in epilepsy research [4]. EEG data analysis using ML and DL techniques has been the subject of numerous studies aimed at identifying and forecasting epileptic episodes [5, 10, 12]. More accurate seizure identification has been made possible by developments in EEG processing techniques, such as improved feature extraction methods, improved artifact removal, and improved time-frequency analysis [6]. About 50 million individuals worldwide suffer from epilepsy, which is one of the most common and dangerous neurological conditions, affecting 1%–2% of the world’s population [7]. Given the severity of seizures and their risks, alongside the pressing demand for more accurate and advanced therapeutic approaches, they have been developed to identify seizure activity and deliver targeted electrical stimulation to help suppress seizures and reduce their frequency [9]. Brain–computer interface (BCI), sometimes known as brain–machine interface (BMI), is a buzzword in the field of neuroscience that involves converting human brain thoughts into a chip. These devices can be surgically implanted or placed externally. Such components enable the user to operate the actuators or detect input data via bilateral communication in order to complete the task [19]. The BCI has been used primarily for rehabilitation, prosthetic control, and neurofeedback [25].

This work solely focuses on the development of hybrid models as well as traditional ML models to find out the potentiality for deployment for aiding patients with epileptic seizures, along with classifying their type and finally providing the aid as needed. The proposed research is divided into various sections, in the “Introduction” section is related to the general introduction along with related works. In the “Materials and methods” section refers to the methodology section in which various ML models are proposed and the proposed architecture is shown. In the “Results and discussions” section shows the results from the models as well as discussions of the results obtained finally. A subsection in results and discussion presents on limitations of the study and future scopes of this research. The final section deals with concluding the study, describing its outcomes and potential application for real-world epilepsy-related effects minimization with AI-powered technology. The work provides a framework for utilizing the Medical Internet of Things (IoMT) for the purpose of early epilepsy detection and aiding patients.

### Related works

In recent years, significant progress has been achieved in employing machine learning (ML) approaches to predict and identify epileptic seizures via EEG readings. Rasheed *et al*. [1] provided a comprehensive overview of cutting-edge machine learning algorithms for early seizure prediction, noting existing research gaps, limitations, and potential future initiatives to improve robustness and generalizability.

Various works utilized the UCI Epileptic Seizure Recognition dataset for classification tasks. Kode *et al*. [2] tested several classifiers, including Extreme Gradient Boosting, TabNet, Random Forest, and a 1D Convolutional Neural Network (CNN), and reported accuracies of up to 99%. Their proposed 1D-CNN outperformed other models in accuracy, sensitivity, precision, and recall. Moving beyond traditional architectures, Zhu *et al*. [3] proposed a multidimensional Transformer combined with recurrent networks (LSTM-GRU) to address the non-stationary and complex nature of EEG signals.

Similarly, Kunekar *et al*. [4] evaluated traditional ML and deep learning (DL) techniques. They discovered that Long Short-Term Memory (LSTM) networks performed better in seizure detection. Vieira *et al*. [5] also proposed an LSTM-based method which could achieve a validation accuracy of 97% while reducing features and channels, demonstrating that lightweight models that use Explainable AI (XAI) can achieve over 95% accuracy with only six features and five EEG channels, making them suitable for mobile applications.

Disli *et al*. [8] introduced a Continuous Wavelet Transform (CWT)-based depthwise CNN that translated multi-channel EEG into image-like representations with about 96% accuracy, exceeding previous methods that did not require further feature extraction or classifiers. The potential of lightweight systems for real-time use has also been investigated.

Tsanika *et al*. [9] developed a TinyML model using iEEG data, achieving up to 99% accuracy, highlighting the feasibility of deploying resource-efficient models in closed-loop neurostimulation devices. Brari *et al*. [10] presented a correlation dimension-based feature extraction method that achieves near-perfect classification accuracy while remaining computationally efficient.

Furthermore, Raab *et al*. [11] combined deep learning with domain knowledge—such as frequency bands, electrode placements, and temporal patterns—to introduce XAI4EEG, a SHAP-based system meant to increase interpretability and reduce validation time for clinicians.

Architectural advances that combine CNNs and recurrent models have also proven extremely beneficial. Mallick *et al*. [12] created a hybrid architecture that included 1D convolutional layers, bidirectional LSTM, GRU, and pooling layers, obtaining up to 100% accuracy for binary seizure detection on the Bonn dataset and strong results in multi-class classification. Esmaeilpour *et al*. [13] employed CNN-based feature extraction, followed by ensemble approaches such as random forests and support vector machines, to detect preictal states on the CHB-MIT dataset, with a sensitivity of 90.76%. Meanwhile, privacy-preserving methods have gained popularity;

Suryakala *et al*. [14] described a Federated Machine Learning (FML) method for training decentralized EEG data models, which achieved high sensitivity (98.24%) and specificity (99.23%) while maintaining patient data security. Collectively, these works demonstrate the range of techniques—from traditional machine learning and deep learning architectures to explainable and privacy-preserving methods—that are improving the reliability and feasibility of EEG-based seizure identification.

To solve the limitations of reliable keyboard-BCI, Paneru *et al*. [18] proposed an EEG-based BMI system that accurately identifies voluntary keystrokes via right- and left-hand movements. The study preprocessed EEG data using band-pass filtering, segmented them into 22-electrode arrays, and refined them into event-related potential (ERP) windows. The resulting 19 × 200 feature array was classified into three categories: resting state, “d” key press, and “l” key press. Recognizing the limits of traditional models, the researchers used a hybrid deep learning architecture, BiGRU-Attention, to efficiently interpret the EEG signals.

Su *et al*. [22] proposed the Epileptic EEG Detection and Recognition Model based on Multiple Attention Mechanisms and Spatiotemporal Feature Fusion (MASF), which aimed to improve the accuracy of epilepsy detection using EEG signals. Recognizing the limitations of CNNs in capturing global dependencies and LSTMs in dealing with long sequences, the authors combined a hybrid attention mechanism, Transformer encoder, and dot-product attention to extract both spatial and temporal dependencies directly from raw EEG data, eliminating the need for complex preprocessing or handcrafted feature extraction. Under 10-fold cross-validation, the proposed MASF model attained outstanding accuracies of 94.19% on the CHB-MIT dataset and 72.50% on the Bonn University dataset, demonstrating its resilience and generalizability. Table 1 compares the proposed method with recent high-performing approaches on benchmark EEG datasets.

**Table 1:** Related studies for seizure detection.

Reference	Method	Dataset used	Result/performance
**Kashefi Amiri *et al*. [26]**	DWT → 1D CNN → LSTM → Fully connected layer	TUSZ, BONN, CHB-MIT	TUSZ: 94.32% accuracy, 86.08% Kappa, 79.01% GDRBONN: 97.24% accuracy, 97.92% Kappa, 99.18% GDRCHB-MIT: 96.94% accuracy, 94.33% Kappa, 96.36% GDR
**Xu *et al*. [27]**	1D CNN → LSTM → Fully connected layers	UCI EEG dataset	Binary: 99.39% accuracyFive-class: 82.00% accuracy
**Cao *et al*. [28]**	DWT → Feature extraction → SVM-RFE → CNN-BiLSTM	Bonn, New Delhi, CHB-MIT	Binary: 100% accuracy, sensitivity, specificity, precision, F1-scoreThree-class (Bonn): 96.19% accuracy, 95.08% sensitivity, 97.34% specificity, 97.49% precision, 96.18% F1-scoreCHB-MIT: 98.43% accuracy, 97.84% sensitivity, 99.21% specificity, 99.14% precision, 98.39% F1-score
**Torkey *et al*. [29]**	CNN → LSTM → GRU → Data balancing → XAI (SHAP)	EEG datasets (MIoT)	Highest accuracy: 99.13%
**Payne *et al*. [30]**	CNN → LSTM for seizure forecasting	NeuroVista dataset	Predicted seizure onset for multiple window sizes (2–40 min); improved patient-specific seizure forecasting over random predictor
**Batista *et al*. [31]**	Feature extraction → SVM + Post-processing	EPILEPSIAE database (TLE patients)	Cumulative Firing Power approach: 62% of patients above chance; Control: 49% above chance
**Esmaeilpour *et al*. [13]**	CNN → Feature extraction → Multiple classifiers → Maximum voting	CHB-MIT	Sensitivity: 90.76% for preictal segment detection
**Ahmad *et al*. [34]**	1D CNN → BiLSTM (TBPTT) → SMOTE → Softmax & Sigmoid	UCI EEG dataset	High precision, sensitivity, specificity, F1-score; multi- and binary-class tasks
**Srinivasan *et al*. [33]**	3D Deep Convolutional Auto-Encoder → BiLSTM classifier	CHB dataset	Accuracy: 99.08 ± 0.54%, Sensitivity: 99.21 ± 0.50%, Specificity: 99.11 ± 0.57%, Precision: 99.09 ± 0.55%, F1-score: 99.16 ± 0.58%
**This work**	Multi-Dimensional CNN-bi-GRU model and IoT implementation for seizure alert	Mendely dataset	Accuracy of 97%+. Alert delivering via BCI application to IoT system and enabling automation of patient surrounding in case of seizure detection.

Despite significant advances as seen in Table 1, further research is required to address the diversity of seizure occurrences and to strengthen early detection frameworks. The emergence of the Internet of Medical Things (IoMT) has introduced promising assistive strategies for enhancing patient care and overall quality of life. While previous studies have often evaluated hybrid deep learning frameworks using only binary or four-class classifications [5], research on three-class seizure detection remains limited. In this study, we focus on detecting various seizure types by first comparing different baseline models against the proposed MDCBG model on the Mendeley dataset. We then implement it alongside both traditional and state-of-the-art hybrid deep learning models to classify multiple seizure types. A multi-dimensional CNN Bi-GRU model is implemented that is used to classify various types of seizures. It is compared with baseline EEGNet and CTNet, and an ablation study is also carried out for the proposed model.

Finally, an IoMT-based framework has the potential to automate patient room and environmental monitoring while providing real-time emergency alerts to caregivers, thereby bridging clinical efficacy with possibility for practical deployment. For this, a tkinter application interface is developed to simulate model predictions on testing set data, and for seizure detection on test data (new data), the notification is also sent to the registered user through the IoT system and SMTP (Simple Mail Transfer Protocol).

## Materials and methods

### Dataset description

A preprocessed dataset from the Mendeley source is collected, and no exclusive filtering methods are applied, as the dataset was pre-filtered [15].

### Preprocessing and feature extraction

The preprocessing of EEG data here in the pipeline begins by loading raw.edf files for six patients (Patients 10 through 15), with each patient’s dataset consisting of multiple EEG recordings. The EEG files are read using the MNE library with preload as “True” to allow in-memory processing. For consistent channel configuration, certain channels are either dropped or reordered depending on the patient ID to ensure uniformity across all recordings [15].

Once the data is loaded, seizure annotations are extracted using a custom Seizure_times module. Each seizure’s start time and duration are translated from real-time to sample indices using a sampling rate of 500 Hz. Based on these indices, seizure segments are sliced from the raw EEG matrix. These seizure segments are stored separately into three categories—CPS, electrographic, and video-detected [15]. To balance the dataset, normal (non-seizure) EEG segments of equivalent length are also extracted from the parts of the signal that do not overlap with any seizure events. These normal and seizure segments are then broken into 1-second epochs (i.e. 500 samples per channel) and stored as 3D arrays with shape (epochs, channels, samples).

All EEG epochs were normalized using z-score standardization, where the mean and standard deviation were computed from the training set and then applied to both training and test data [21]. This ensured that the data achieved zero mean and unit variance, producing standardized EEG epochs that were ready for input into all machine learning models.

*Equations:*

For each element $x_{i,c,t}$ in the 3D tensor $x\in R^{N\times C\times T}$ (samples × channels × timepoints):


(1) $$\frac{1}{N\cdot C\cdot T}\sum_{i=1}^{N}.\sum_{c=1}^{C}.\sum_{t=1}^{T}.x_{i,c,t}$$

(2) $$\sigma =\sqrt{\frac{1}{N\cdot C\cdot T}\sum_{i=1}^{N}.\sum_{c=1}^{C}.\sum_{t=1}^{T}.{\left(x_{i,c,t}-\mu \right)}^{2}}$$

Then, normalized training data:


(3) $${\tilde{x}}_{i,c,t}=\frac{x_{i,c,t}-\mu }{\sigma }$$

And test data is normalized using the training mean & std:


(4) $${\tilde{x}}_{i,c,t}^{\mathrm{test}}=\frac{x_{i,c,t}^{\mathrm{test}}-\mu }{\sigma }$$

The preprocessed EEG epochs were labeled and stacked into a single feature matrix x, with labels as follows: 0 for normal, 1 for CPS, 2 for electrographic. Since class 3 is a minority class with very few samples, it was excluded from the training pipeline. The final dataset was split into training and testing sets using a 90:10 ratio, and all arrays were saved in.npy (NumPy) format to support flexibility in further analysis and model training. The dataset labels are summarized in Table 2, and the dataset size is provided in Table 3.

**Table 2:** Labels on dataset.

Label	Description
**0**	Normal EEG data (non-seizure)
**1**	Complex Partial Seizures
**2**	Electrographic Seizures
**3**	Video-detected Seizures (no EEG visible change)

**Table 3:** Dataset size.

Dataset	Class 0	Class 1	Class 2	Class 3	Total
**Train**	3479	2745	682	105	7011
**Test**	416	289	68	6	779

The distribution of samples by class in the training and testing datasets is summarized in Table 3. Classes 0 and 1 make up the majority of the 7011 samples in the training set, whereas Classes 2 and 3 are notably underrepresented, suggesting a clear class imbalance. The test set, which comprises 779 samples, shows a similar distribution, with relatively few instances of Class 3. To compare the performance of the suggested model with baseline techniques, the dataset is first used for multiclass classification by classifying Class 0 as non- seizure and merging all other classes as seizure. As discussed earlier, due to the extremely limited number of samples in Class 3, this class is excluded from further analysis, and only Classes 0, 1, and 2 are considered for seizure-type classification.

**Figure 1:**
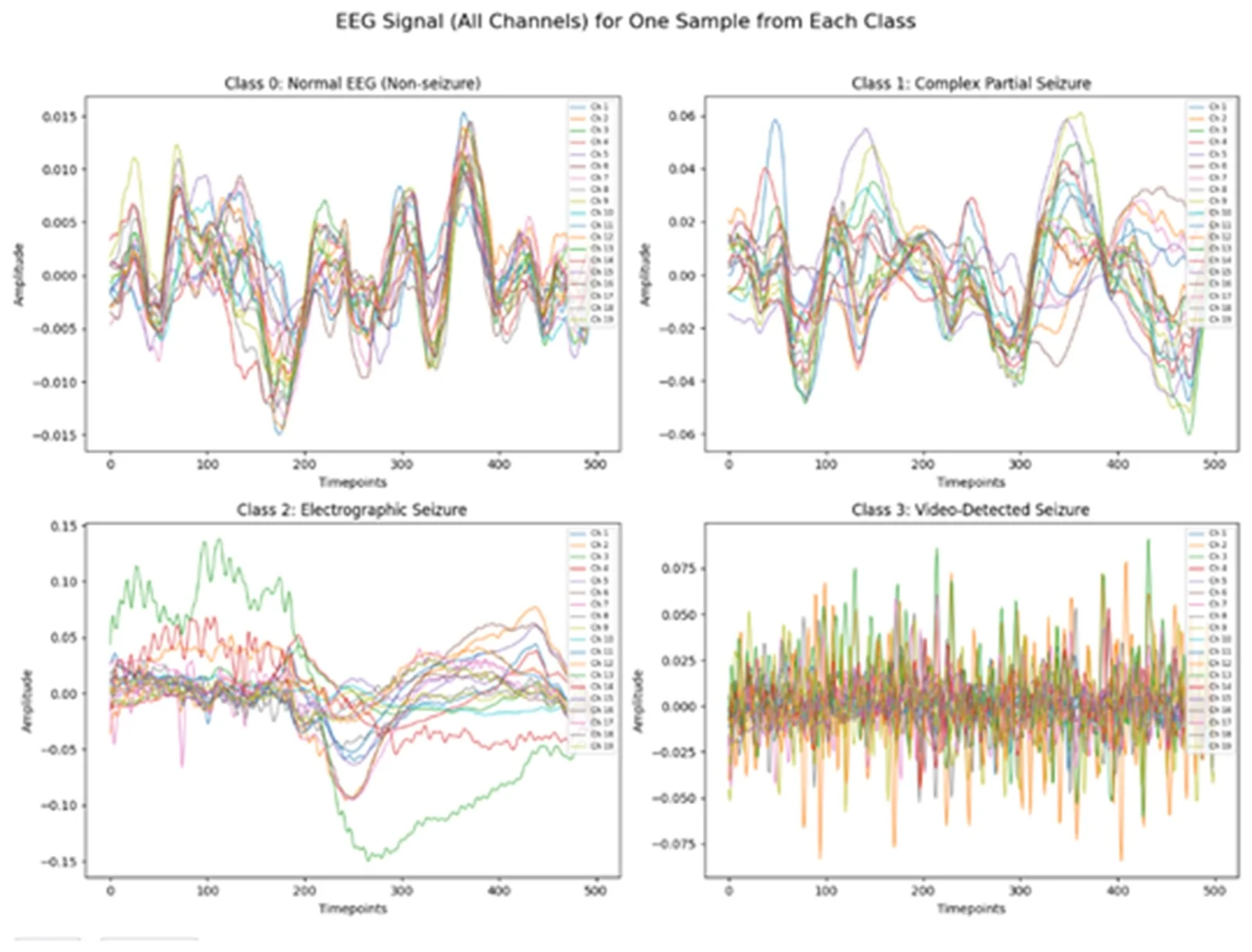
EEG signal variation plot for various events.

**Figure 2:**
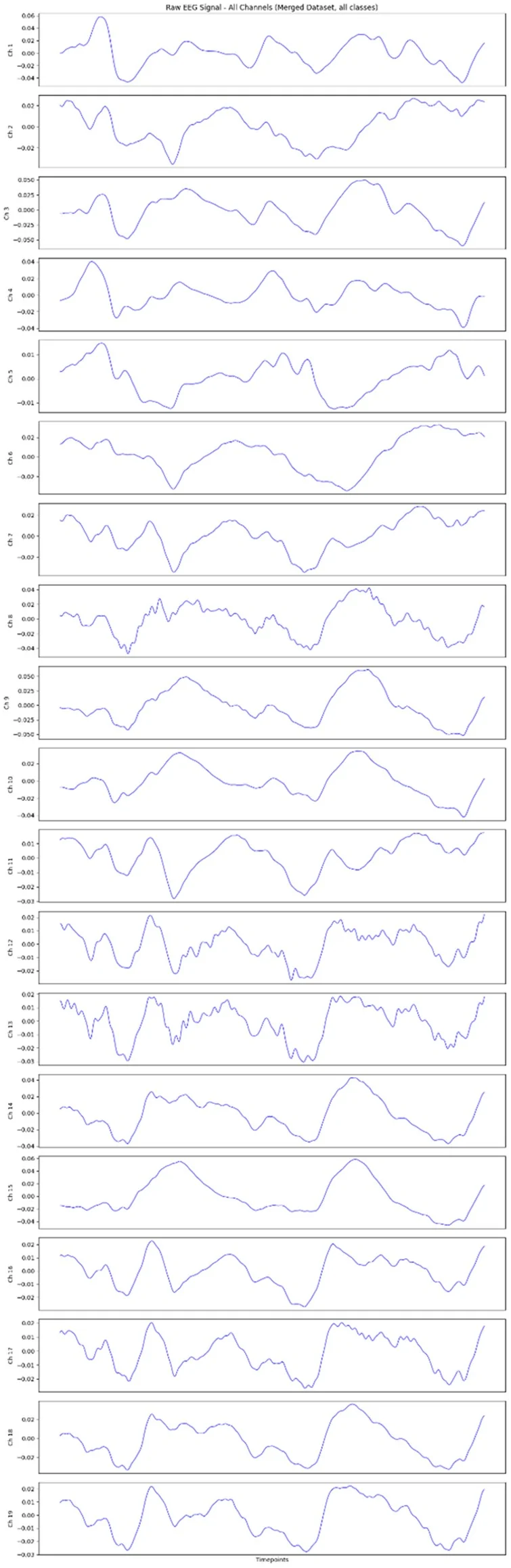
EEG data plot for all channels.

The EEG plots in Fig. 1 shows representative signals across all 20 channels from each of the four classes. Class 0 (Normal EEG) shows low-amplitude, rhythmic waveforms without significant abnormalities, reflecting non-seizure brain activity. Class 1 (Complex Partial Seizure) exhibits moderate amplitude fluctuations and irregular patterns, indicating localized seizure onset. Class 2 (Electrographic Seizure) displays prominent amplitude shifts and gradual trends, characteristic of seizures detected solely through EEG. In contrast, Class 3 (Video-Detected Seizure) shows high-frequency noise-like patterns, possibly indicating seizure activity visible externally (e.g. motor movements) but less prominent in EEG amplitude. The channel-wise variations can be visualized from Fig. 2.

### EEG 10–20 system

**Figure 3:**
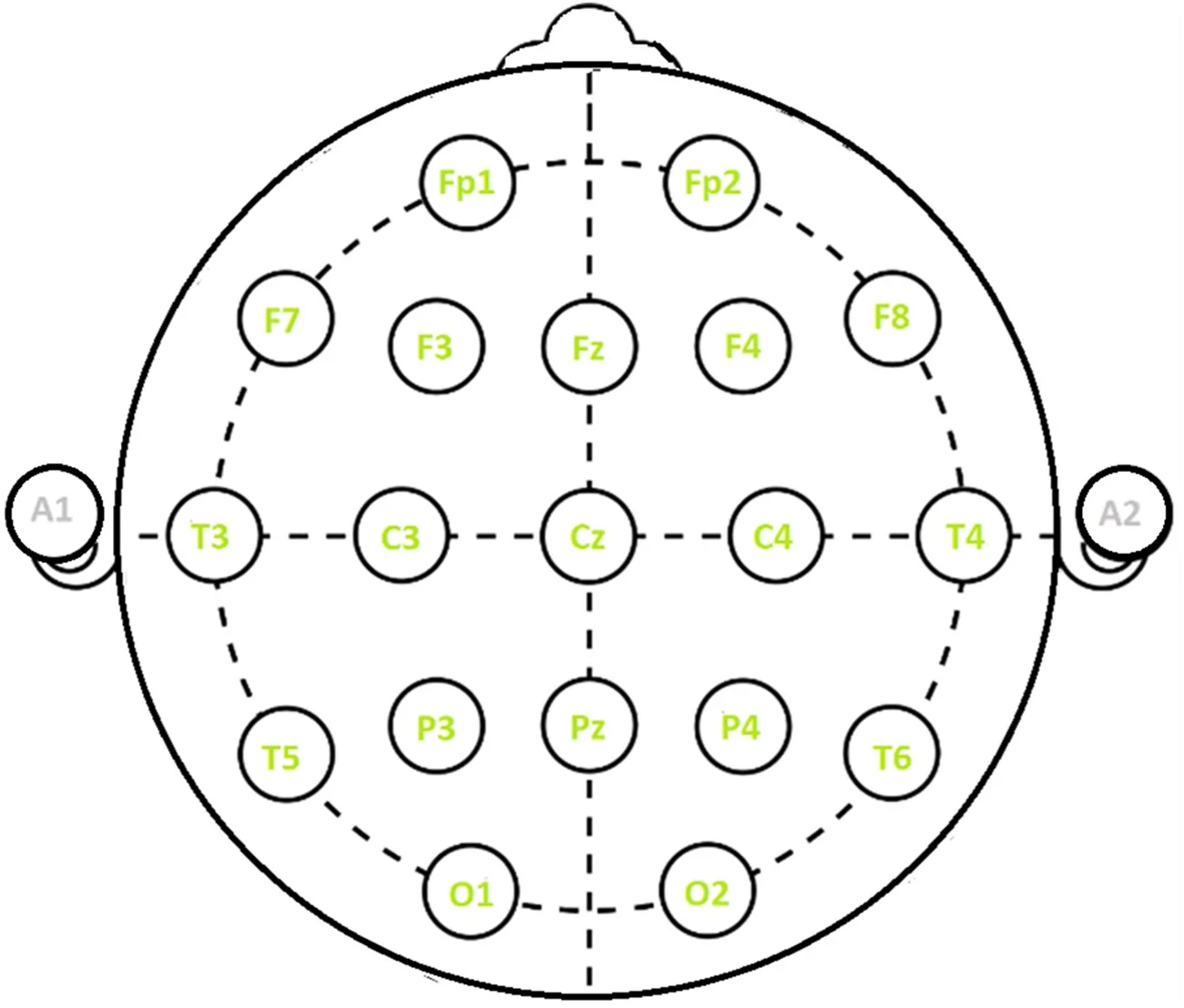
10–20 electrode system.

A widely accepted method for placing electrodes on the scalp to record electroencephalogram (EEG) data is the typical 10–20 electrode system, as seen in Fig. 3 [24]. The electrode placement is referred to as “10–20” and takes place at intervals of 10% or 20% of the total distance between specific anatomical landmarks on the skull, such as the nasion (front), inion (back), and preauricular points (sides).

**Figure 4:**
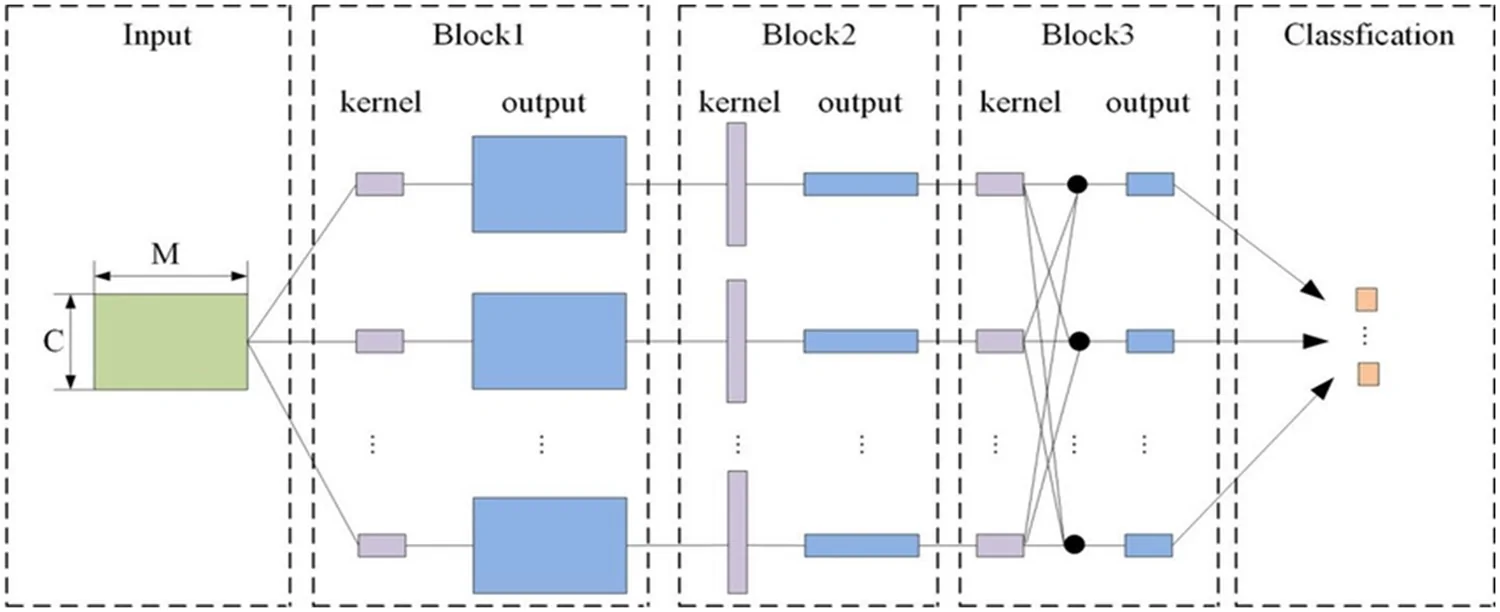
EEGNet architecture [16].

**Figure 5:**
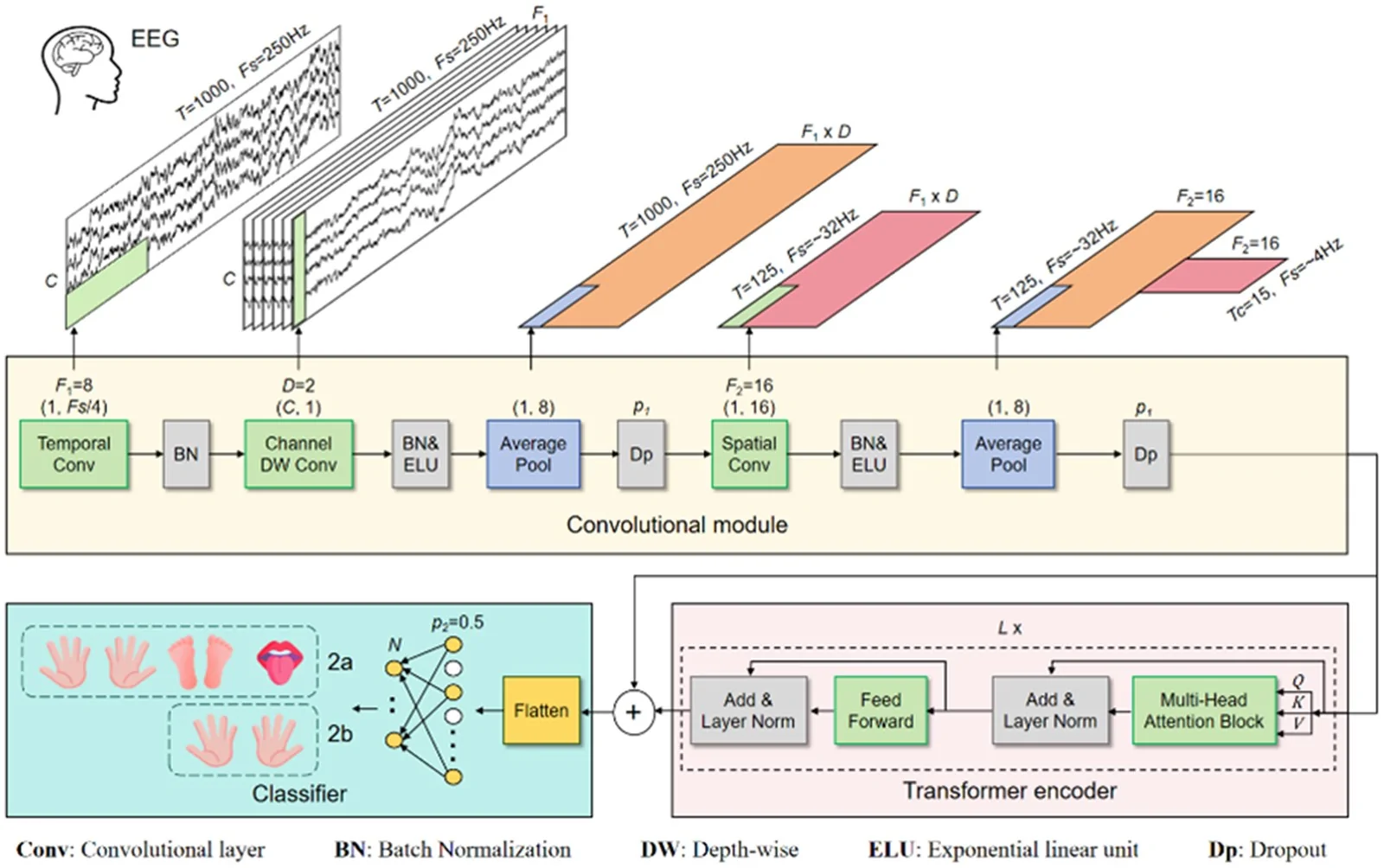
CTNet architecture [34].

Electrodes are labeled with letters that represent the area of the brain (F for frontal, T for temporal, P for parietal, O for occipital, and C for central) and numbers that represent the locations of the left (odd numbers) or right (even numbers) hemispheres. In neurophysiology, clinical diagnosis, and research, the 10–20 system is widely used to record brain rhythms, detect abnormalities such as epilepsy, and examine cognitive functions.

### Models utilized

#### Bi-Directional LSTM GRU

This model combines Bi-Directional Long Short-Term Memory (LSTM) and Gated Recurrent Unit (GRU) layers to capture temporal dependencies in sequence data from both past and future contexts. The bi-directional setup allows the network to understand patterns from forward and backward sequences, improving performance on time-series tasks like EEG signal classification.

#### Extra trees

Extra Trees (Extremely Randomized Trees) is an ensemble learning method that builds multiple decision trees using random splits of features and thresholds. Unlike Random Forests, Extra Trees inject more randomness, often leading to faster training and sometimes better generalization. It is effective for tabular data classification and regression tasks.

#### Bi-Directional GRU with attention

This model enhances a bi-directional Gated Recurrent Unit (GRU) network by incorporating an attention mechanism that allows the model to focus on important time steps in the sequence. The attention layer improves interpretability and performance by weighting relevant parts of the input, which is particularly useful for complex sequential data such as EEG signals [18].

#### Baseline models (EEGNet)

EEGNet is a tiny convolutional neural network designed specifically for EEG signal analysis, with a focus on efficiency and physiological interpretability. Depthwise spatial convolutions are utilized to function as data-driven spatial filters across EEG channels and record inter-channel interactions after temporal convolutions have been used to extract frequency-specific features from EEG time series. Thus, separable convolutions improve temporal patterns while drastically reducing the number of parameters. The network uses batch normalization, ELU activations, average pooling, and dropout to stabilize training and prevent overfitting. EEGNet is computationally efficient and can function well even with minimal EEG data because to its lightweight design [16]. The models architecture is shown in Fig. 4.

#### CTNet model

CTNet, developed by Zhao *et al*. [34], is a convolutional Transformer network designed for EEG-based motor imagery (MI) classification. It combines a convolutional module, inspired by EEGNet, to extract local temporal and spatial features from EEG signals with a Transformer encoder employing multi-head attention to capture global dependencies in the high-level feature representation. The model concludes with a fully connected classifier that maps the combined features to MI categories. CTNet achieves competitive decoding accuracies in both subject-specific and cross-subject evaluations, demonstrating the effectiveness of integrating convolutional feature extraction with Transformer-based attention for robust EEG signal classification.

The architecture of CTNet shown in Fig. 5 consists of three main components. First, the convolutional module encodes low-level spatial–temporal features from EEG trials using temporal, depth-wise, and spatial convolutions, followed by batch normalization, ELU activation, average pooling, and dropout to generate compact feature maps. Second, the Transformer encoder processes these feature maps as sequential tokens, employing multi-head attention and position-wise feed-forward layers with residual connections and layer normalization to capture global temporal dependencies. Finally, the classifier module combines the convolutional and Transformer features, applies dropout, flattens the result, and passes it through fully connected layers to output the MI class probabilities. This design enables end-to-end EEG decoding without handcrafted feature extraction, effectively integrating local and global signal information [34].

#### MDCBG (proposed model)

**Figure 6:**
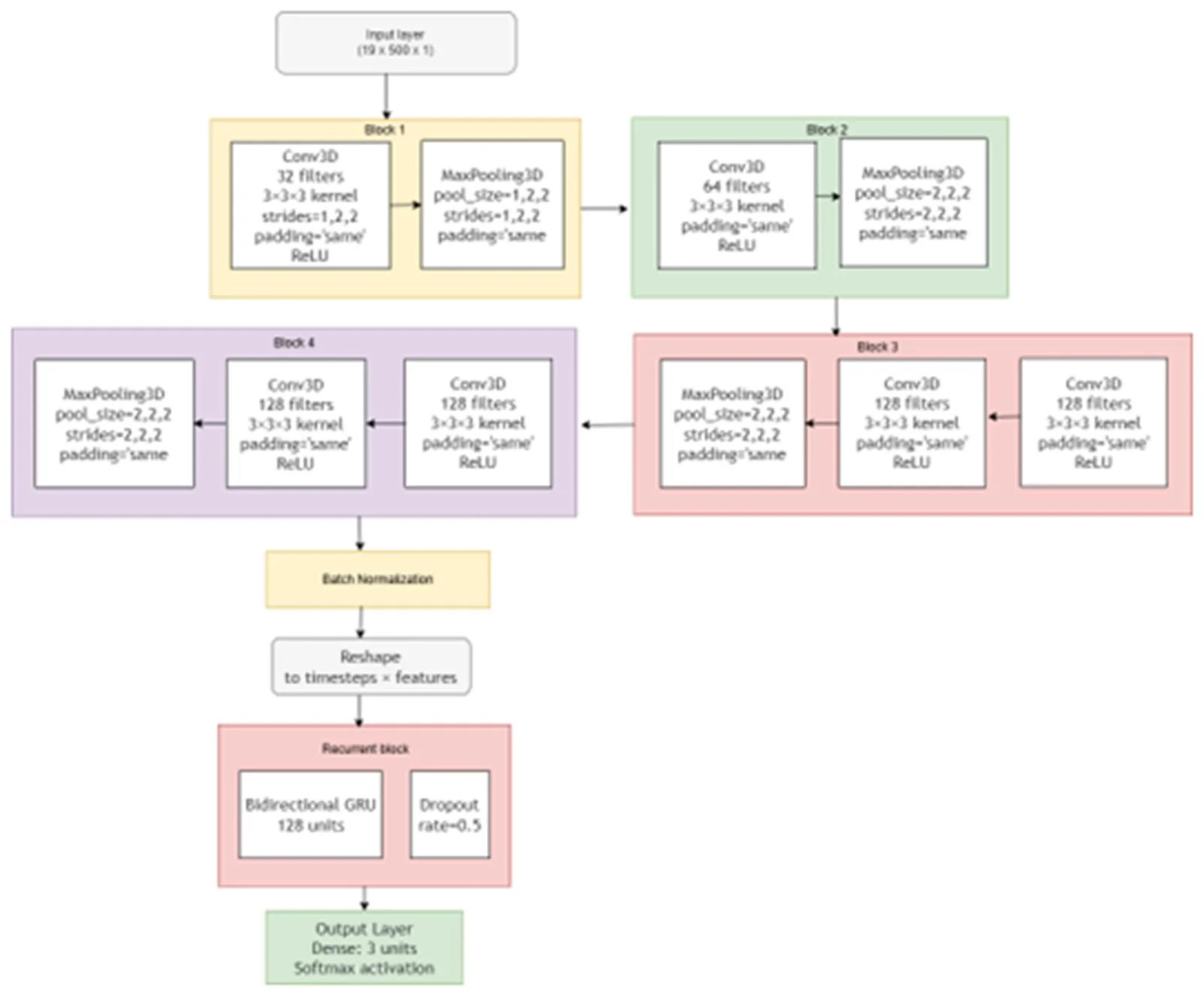
Proposed multi-dimensional CNN-BI-GRU model architecture.

This model architecture in Fig. 6 is a hybrid Multi-Dimensional CNN + Bi-GRU (MDCBG) architecture designed for binary EEG classification (seizure vs. non-seizure). The input EEG data, with shape (19 channels × 500 time samples), is first reshaped for compatibility with 3D convolutional layers, allowing the model to capture both spatial (across channels) and temporal (across time) patterns simultaneously. The network consists of four convolutional blocks: the first uses 32 filters with a (3 × 3 × 3) kernel and stride (1,2,2) followed by 3D max pooling to reduce spatial dimensions; the second block increases the filters to 64, again followed by max pooling; and the third and fourth blocks each have two consecutive Conv3D layers with 128 filters, capturing deeper hierarchical features, with max pooling after each block. Batch normalization is applied to stabilize and accelerate training.

The resulting feature maps are flattened along the spatial dimensions and fed into a Bidirectional GRU, which models temporal dependencies in both forward and backward directions. A dropout layer prevents overfitting, and a final dense layer with a sigmoid activation outputs the binary prediction. This combination of 3D convolutions for spatiotemporal feature extraction and BiGRU for temporal sequence modeling makes the model particularly effective for EEG-based seizure detection.

#### Random Forest

Using various bootstrap samples of the data and random subsets of characteristics, Random Forest is an ensemble learning technique that creates several decision trees. By combining the results of all trees (majority voting or average), the final forecast is produced, which lessens overfitting, increases robustness, and is still rather simple to understand and adjust.

#### XGBoost

XGBoost (Extreme Gradient Boosting) is a powerful boosting-based ensemble technique where trees are built sequentially, and each new tree focuses on correcting the errors made by the previous ones. It incorporates gradient-based optimization, regularization, and efficient parallel computation, allowing it to achieve high predictive accuracy and strong generalization, especially on structured and tabular data.

### Proposed workflow

First, dataset was preprocessed and feature extraction was done on the dataset; they are trained and evaluated with various evaluation metrics. In order to find out the best performance, the nested cross-validation technique is applied. This enhanced the model development as well as ensured the model’s performance is not compromised on different folds of the dataset. With this cross-validation technique, model performance on the entire dataset can be rigorously tested. Then, the trained model is proposed to be deployed on the proposed IoMT system-based concept to aid patients.

**Figure 7:**
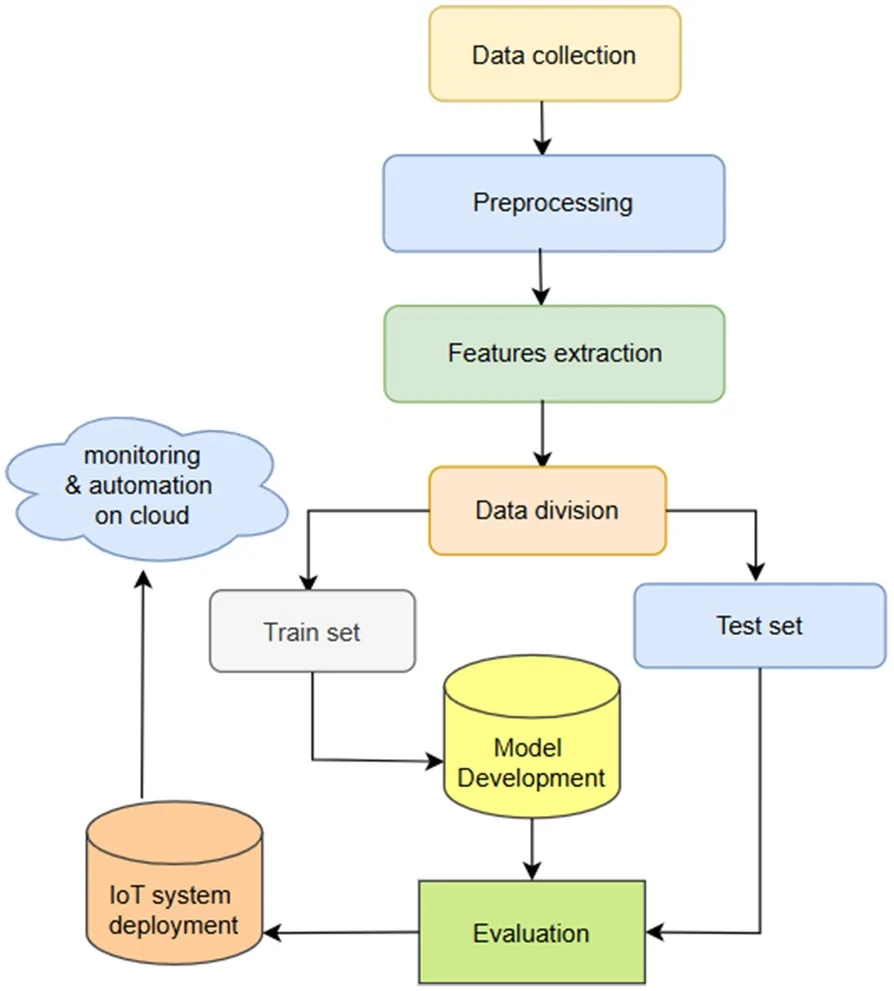
Proposed modeling concept.

**Figure 8:**
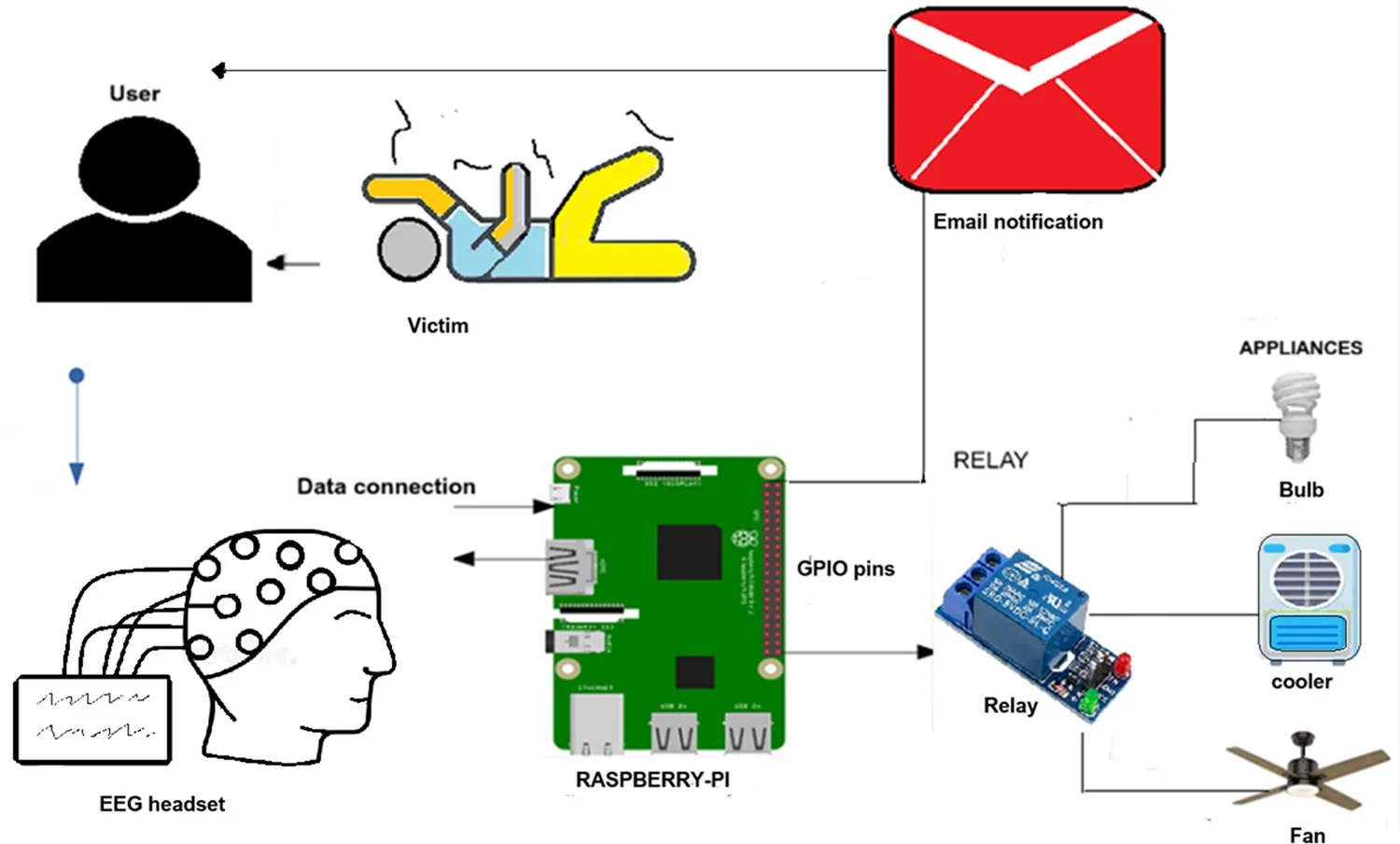
Proposed system concept.

The various machine learning models were trained for seizure detection, as illustrated in Fig. 7. Different hybrid models, are included alongside EEGNet, a widely used model in the EEG analysis field. The complete workflow is shown in Fig. 8. The dataset consists of EEG segments, each of size 19 channels × 500 time points (1 second at 500 Hz). All neural network models were trained on a Kaggle GPU environment with an NVIDIA Tesla P100, with each model requiring approximately 5 minutes for training.

### IoMT-based approach (IoT and EEG interaction for aid)

**Figure 9:**
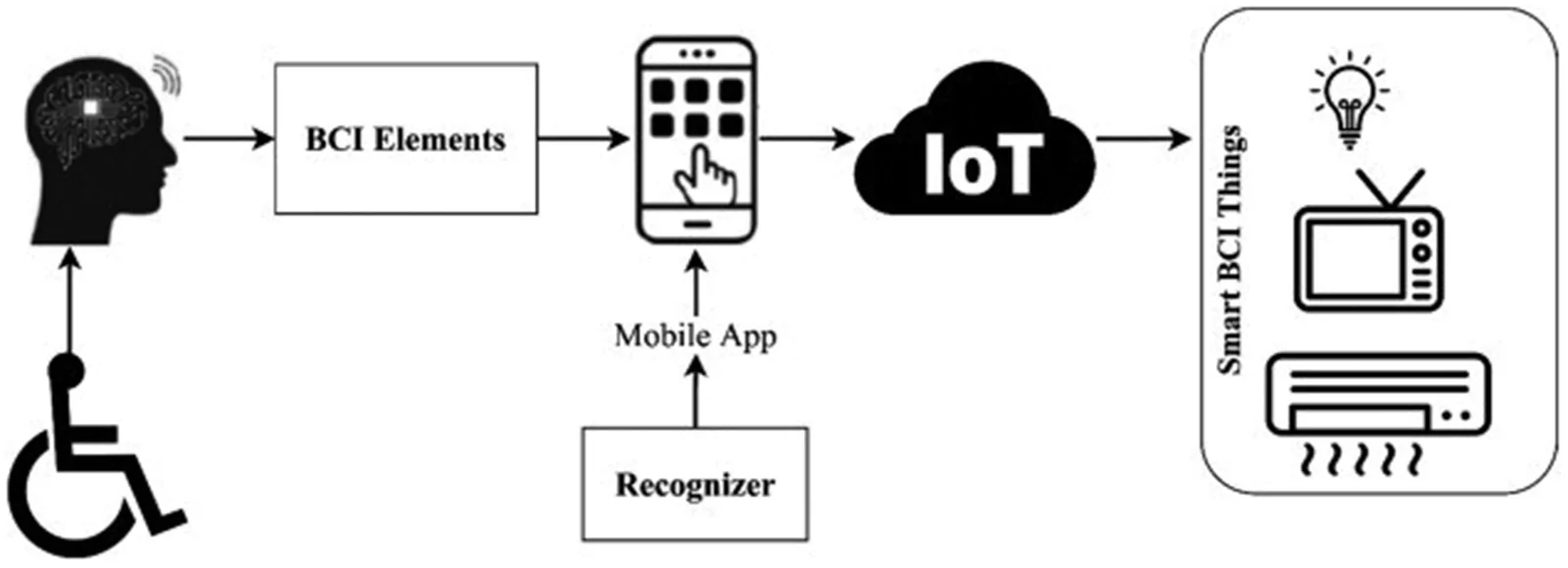
IoT Enabled BCI [19].

The Internet of Things with cloud databases can be used to record the data and provide feedback as a brain machine interface or early aiding for patients. BCIs allow direct communication between the brain and external devices, providing a lifeline for people with significant physical limitations. Incorporating IoT concepts may improve BCI efficacy. The integration of BCI with IoT technology as shown in Fig. 9 offers distinct advantages. BCIs can be linked to the IoT framework to provide a more adaptable and comprehensive communication and control system [20].

In the proposed system (Fig. 8), real-time EEG signals are acquired from the patient via a headset connected to a microcontroller, which continuously streams data to an IoT platform. The data is processed by the trained seizure detection model deployed to the Raspberry Pi microcomputer system, enabling early identification of potential epileptic events. Upon detection, alerts such as emails, SMS, or other notifications can be automatically sent to caregivers or medical personnel to provide timely assistance.

The system also makes it possible to automate the patient’s surroundings. Relay circuits can be used to securely alter room conditions during a seizure by turning on linked appliances like fans, lights, or other gadgets. Real-time inference and incremental learning are supported by the model deployable on the IoT platform, enabling the retraining of freshly collected EEG data to improve predictive performance over time. Continuous monitoring, prompt alarms, and automatic support for epileptic patients are made possible by the integration of an EEG headset, microcontroller, IoT infrastructure, and predictive modeling.

With this concept as in Fig. 8, early aid and automated systems can be made available to facilitate the victim and prevent the deterioration of his/her health further. With early notification, the caretaker can be called early to aid the patient. The IoT system with a low cost developed here consists of an application that is fed with testing set data from a NumPy array, which is sent to a trained pkl model version, which, when it predicts a seizure on test set data (new data), sends the email to the specific user. This system as a proof-of-concept is deployable with lightweight machine learning model conversion compatible to edge or IoT systems with live data acquisition from headset.

### Evaluation metrics

#### Accuracy, precision, recall and F1-score

Accuracy measures the overall correctness of a classification model by calculating the proportion of total predictions that are correct. Precision evaluates how many of the positive predictions made by the model are actually correct, reflecting the model’s ability to avoid false positives. Recall (also known as sensitivity) measures the model’s ability to identify all actual positive cases by finding the proportion of true positives detected out of all real positives, thus indicating how well the model prevents false negatives. The F1-score is the harmonic mean of precision and recall, providing a balanced metric that combines both aspects to obtain one performance measure, especially useful when the class distribution is imbalanced [17, 23].


(5) $$a.\;\mathrm{Accuracy}\;=\;\frac{TN+TP}{\left(FP+FN+TP+TN\right)}$$

(6) $$b.\;\mathrm{Precision}=\frac{TP}{\left(TP+FP\right)}$$

(7) c. Recall= TP/(TP+FN)

(8) $$d.\;F1-\mathrm{Score}=2.\;*\;(\frac{\left(\mathrm{Precision}\;*\;\mathrm{Recall}\right)}{\left(\mathrm{Precision}\;+\;\mathrm{Recall}\right)})$$

#### e. Macro ROC-AUC

It measures how well a multi-class (or imbalanced) classifier separates each class independently, then averages the performance across all classes equally.

For C classes, ROC–AUC is computed in a one-vs-rest manner for each class and then averaged:


(9) $$\text{Macro}\;\text{ROC}–\text{AUC}=\frac{1}{C}\sum_{i=1}^{C}{\text{AUC}}_{i}$$

where ${\text{AUC}}_{i\;}$is the ROC–AUC for class i vs. all other classes.

### Stratified cross validation

A stratified 5-fold cross-validation strategy was employed on the pooled dataset to ensure robust and leakage-controlled model evaluation. In each fold, the dataset was partitioned into mutually exclusive training and testing subsets while preserving the original class distribution through stratification. Data normalization [21] was performed strictly within each fold using statistics (mean and standard deviation) computed only from the training partition, and the same parameters were subsequently applied to the corresponding test partition, thereby preventing any information leakage from test data into the training process. To ensure independent learning across folds, model weights were reinitialized for each fold. Overfitting was further reduced by early halting based on a validation subset that was exclusively obtained from the training data. This approach ensures that every sample is assessed precisely once in an unseen test role, offering a trustworthy assessment of the model’s capacity for generalization and verifying that the stated performance is a reflection of actual learning rather than memorization or data leakage.

To guarantee balanced class representation in each fold, the data was assessed using 5-fold stratified cross-validation. The entire dataset is first split into five mutually exclusive subsets (folds) of roughly equal size in 5-fold stratified cross-validation, with each fold maintaining the original class distribution. Each iteration uses one fold (≈20%) as the test set and four folds (≈80%) as the training set. Each fold is used as the test set exactly once during the five repetitions of this procedure. The stratification technique prevents class imbalance during training and evaluation by ensuring that all folds have proportionate representations of each class. Within each fold, the training data was normalized using z-score normalization, where the fold-specific mean and standard deviation were used to scale both the training and validation sets, preventing any information leakage.

### SHAP analysis

SHAP (SHapley Additive exPlanations) library have been utilized to interpret the model’s predictions on the testing dataset. We start by creating a TreeExplainer object specific to the trained Extra Trees classifier model, which efficiently calculates SHAP values. These SHAP values quantify the contribution of each feature to the prediction for each test sample. Since the problem is multiclass classification, the SHAP values are computed separately for each class, resulting in a list of arrays—one per class.

## Results and discussions

In this section, the performance of multiple models is compared and critically reviewed, along with SHAP-based explanations to identify the most relevant features influencing predictions. The feasibility of incorporating these models into a conceptual framework for early assistance to seizure victims is also discussed, together with the study’s limitations and future research directions.

### Models performance evaluations

#### Binary class (seizure vs no seizure)

##### EEGNet

**Figure 10:**
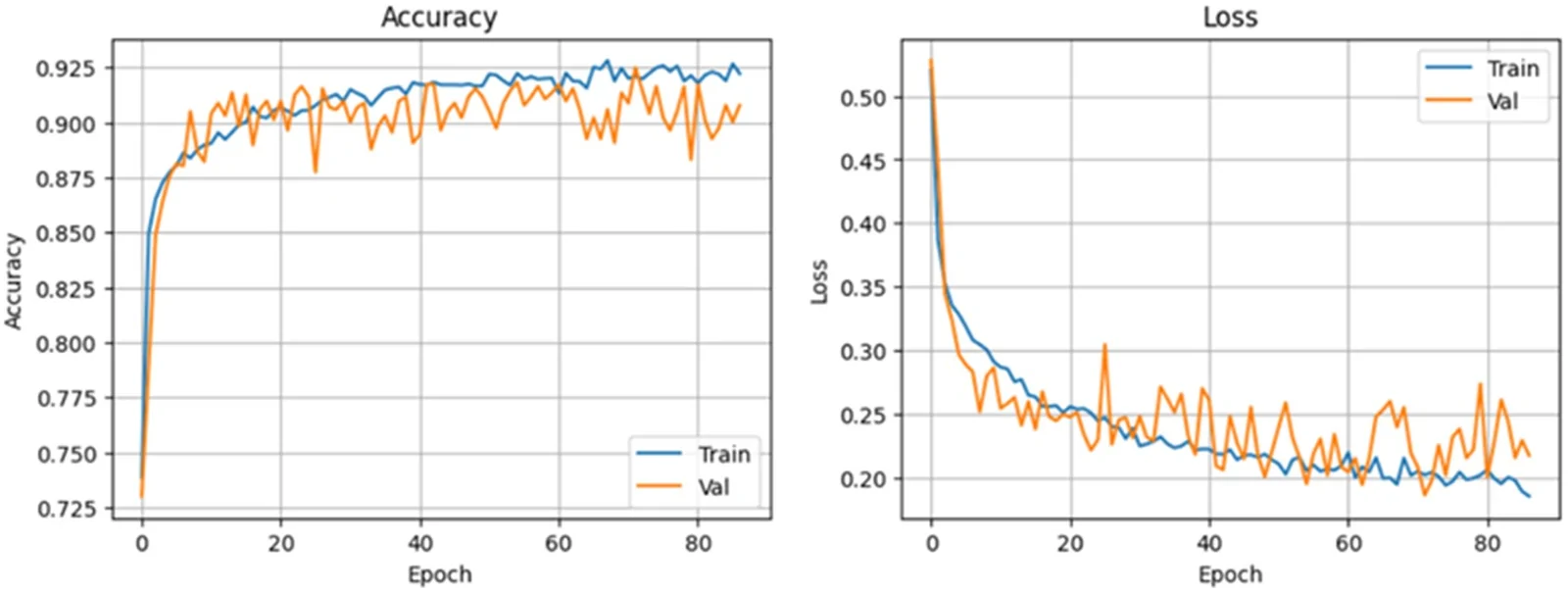
EEGNet History plots.

EEGNet baseline obtained a training Accuracy of 92% including a testing Accuracy of 92.17%. The training history plots in Fig. 10 show that the EEGNet model learns quickly during the early epochs, with both training and validation accuracy rising sharply and stabilizing around 90%–93%, indicating strong and consistent performance.

The validation accuracy closely follows the training accuracy, which suggests that the model is generalizing well without significant overfitting. The loss curves also show a steady decline for both training and validation sets, with validation loss fluctuating slightly due to noise in the data but overall following the same downward trend as training loss. Together, these plots indicate that the EEGNet model is training effectively, achieving good accuracy with stable convergence and no major signs of overfitting or underfitting, as seen in Fig. 10.

**Figure 11:**
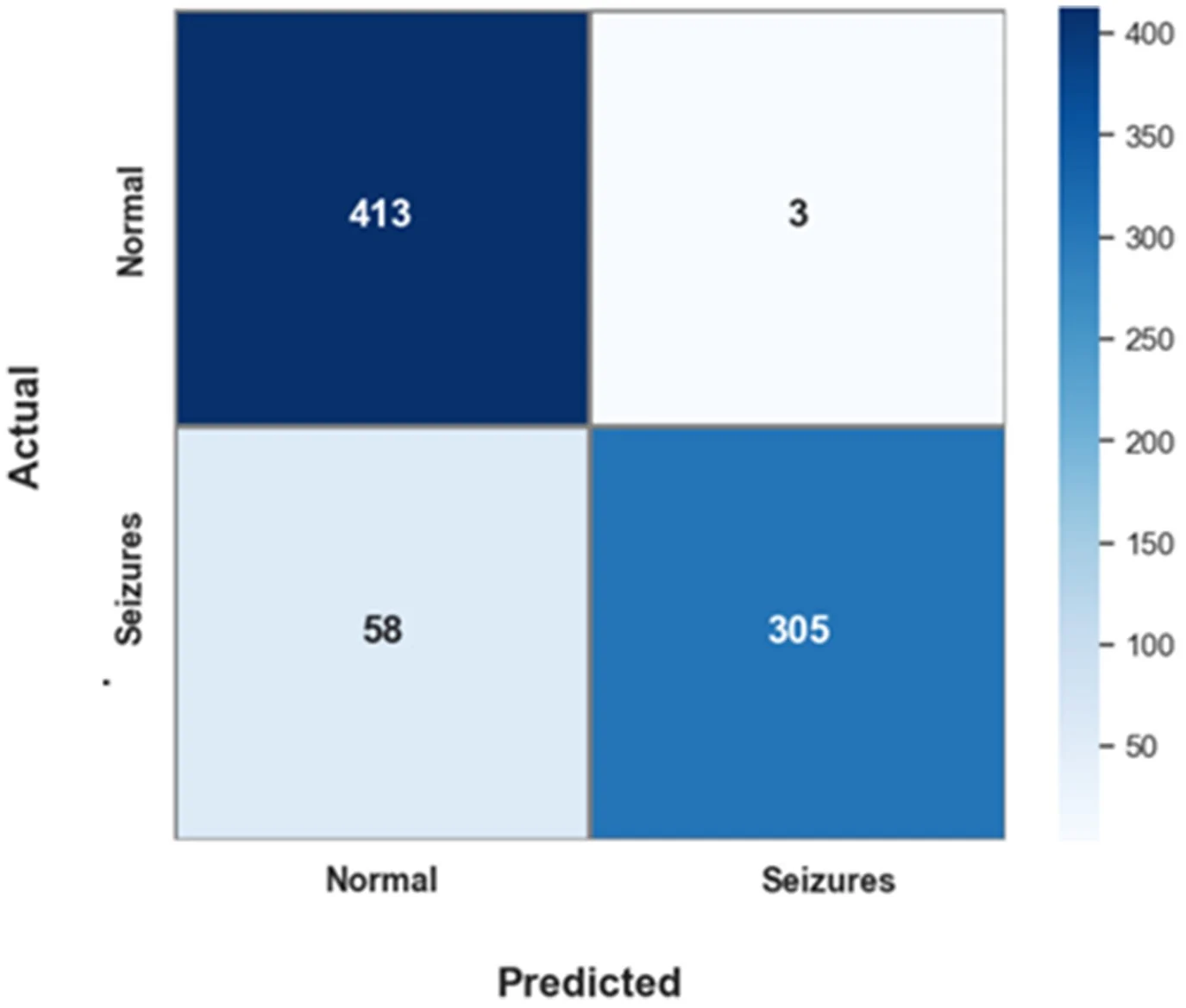
EEGNet confusion matrix plot.

This confusion matrix plot in Fig. 11 shows how well the EEGNet model classified two classes (seizure and no-seizure) labeled 0 and 1. For class 0, the model performed extremely well: out of all actual class-0 samples, it correctly predicted 413 as class 0 and misclassified only 3 as class 1. This means class-0 recall is very high, indicating the model rarely misses class-0 instances. For class 1, the model correctly predicted 305 samples as class 1 but misclassified 58 as class 0. Although the model still performs strongly on class 1, the higher number of false negatives (58) shows that class-1 recall is lower compared to class 0. Overall, the matrix indicates that EEGNet has good accuracy with strong performance on both classes, slightly favoring class 0 due to fewer misclassifications.

##### CTNET baseline

**Figure 12:**
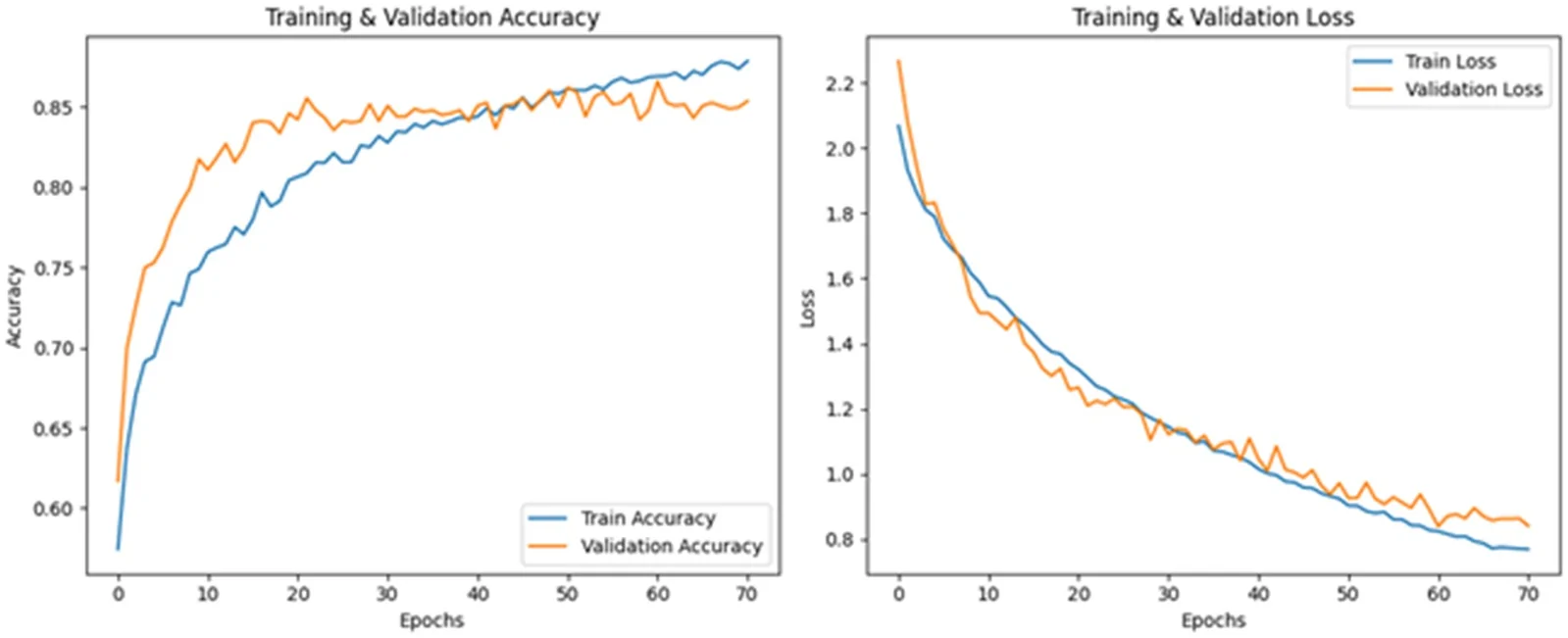
CTNet history plots.

The model obtained a training accuracy of 87.68% and a test accuracy of 85.11%. The training history plots in Fig. 12 show that the CTNet model learns quickly during the early epochs, with both training and validation accuracy rising sharply and stabilizing around 85%–87%, indicating acceptable performance.

The validation accuracy closely follows the training accuracy, which suggests that the model is generalizing well without significant overfitting. The loss curves also show a steady decline for both training and validation sets, with validation loss fluctuating slightly due to noise in the data but overall following the same downward trend as training loss. Together, these plots indicate that the CTNet model does not reaches more that 86%–88% on training and lies within 85% on testing which shows its lack of ability to capture temporal EEG patterns as seen in Fig. 12.

**Figure 13:**
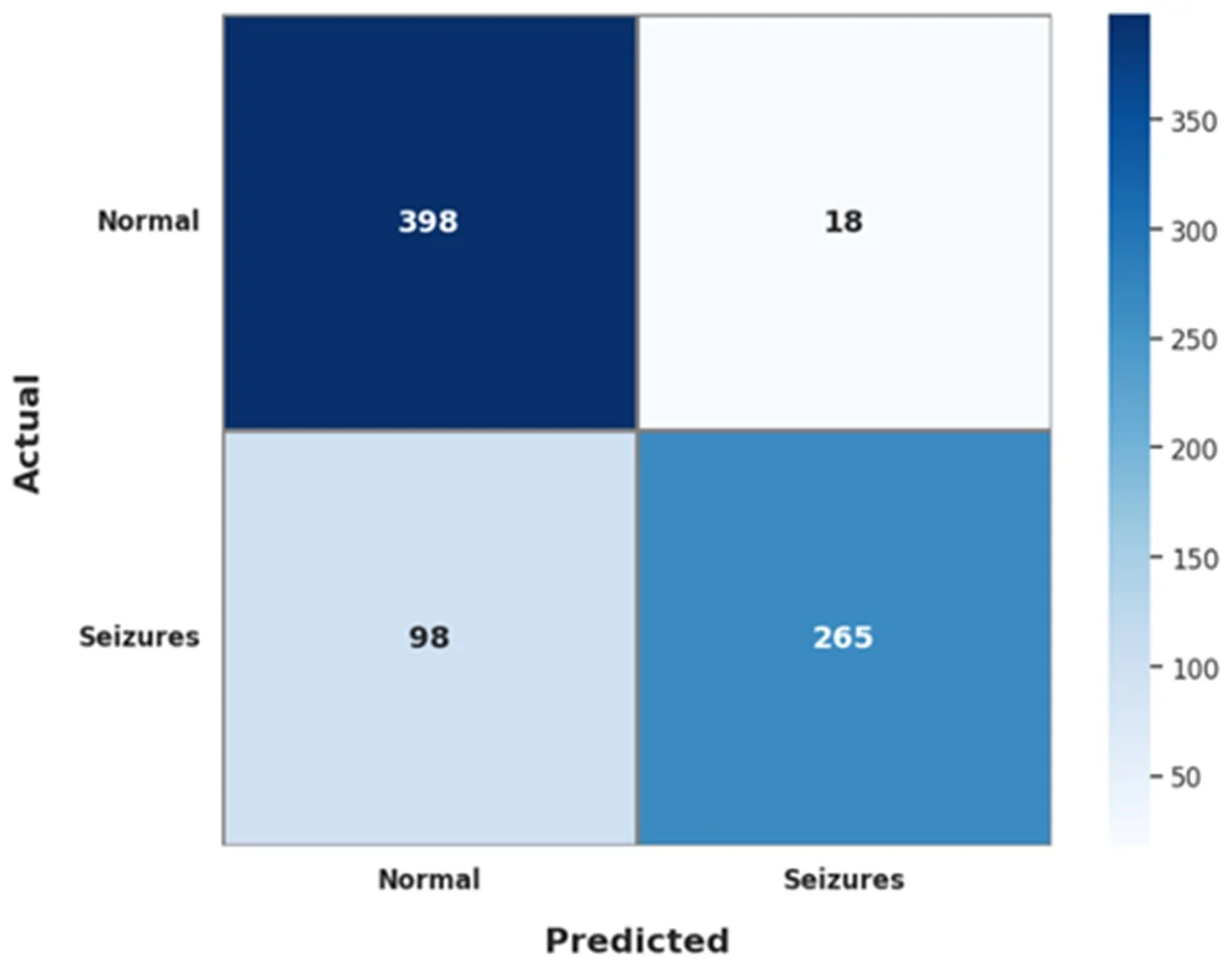
CTNet confusion matrix plot.

This confusion matrix plot in Fig. 13 shows how well the CTNet model classified two classes (seizure and no-seizure) labeled 0 and 1. For class 0, the model performed extremely well: out of all actual class-0 samples, it correctly predicted 398 as class 0 and misclassified 18 instances as class 1. This means class-0 recall is very high, indicating the model rarely misses class-0 instances. For class 1, the model correctly predicted 265 samples as class 1 but misclassified 98 samples. This shows that the baseline CTNet model fails to capture EEG seizure victims’ data patterns and does not learn well on the test set.

##### MDCBG

**Figure 14:**
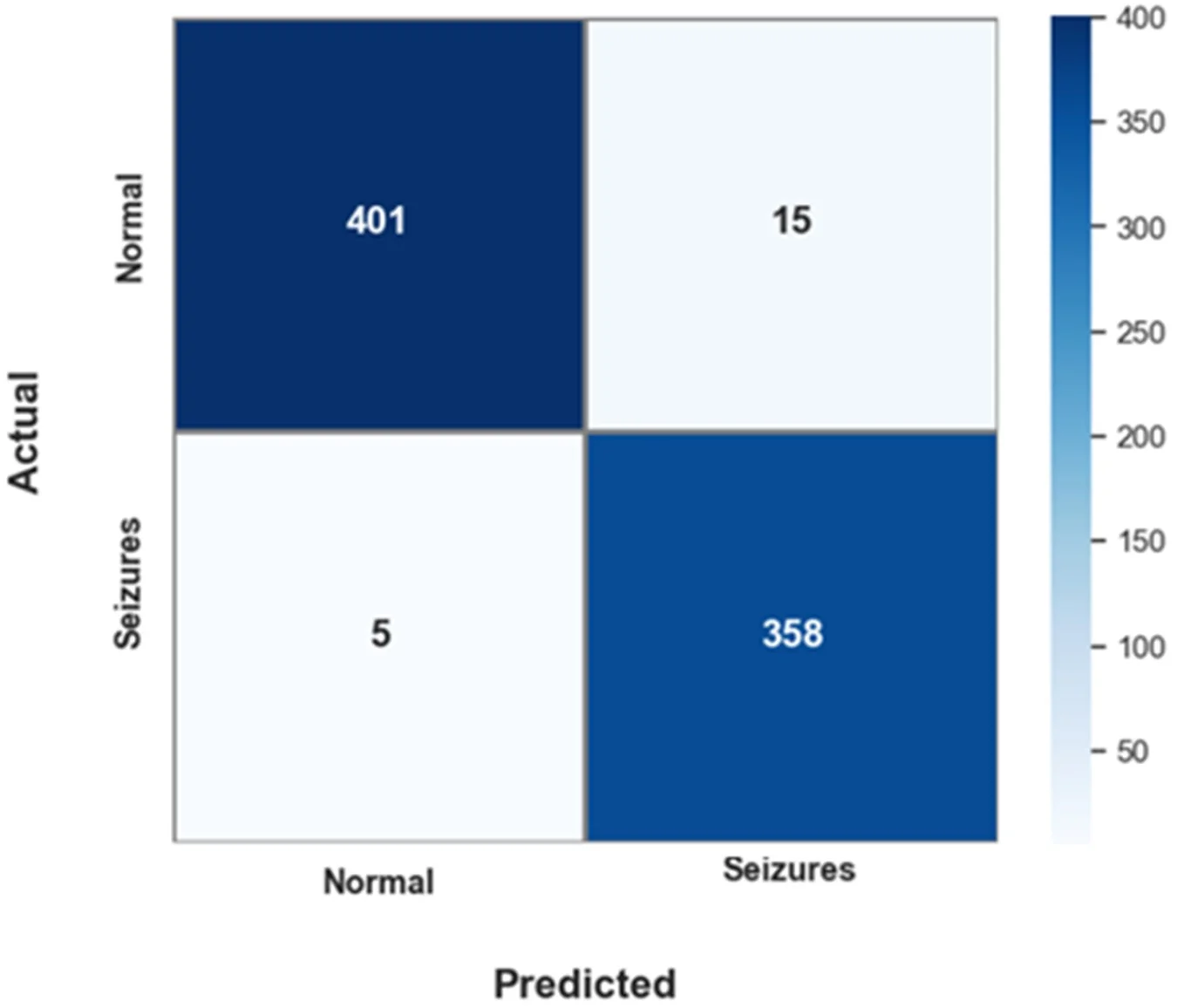
Confusion matrix plot for MDCBG.

The model MDCBG obtained a test accuracy of 97.43% including train accuracy of 99.47%. The confusion matrix plot (Fig. 14) shows that for class “0” 401 instances or testing feature set were correctly classified similarly, for class “1” 358 instances were correctly classified as class “1” as seen in Fig. 14.

**Figure 15:**
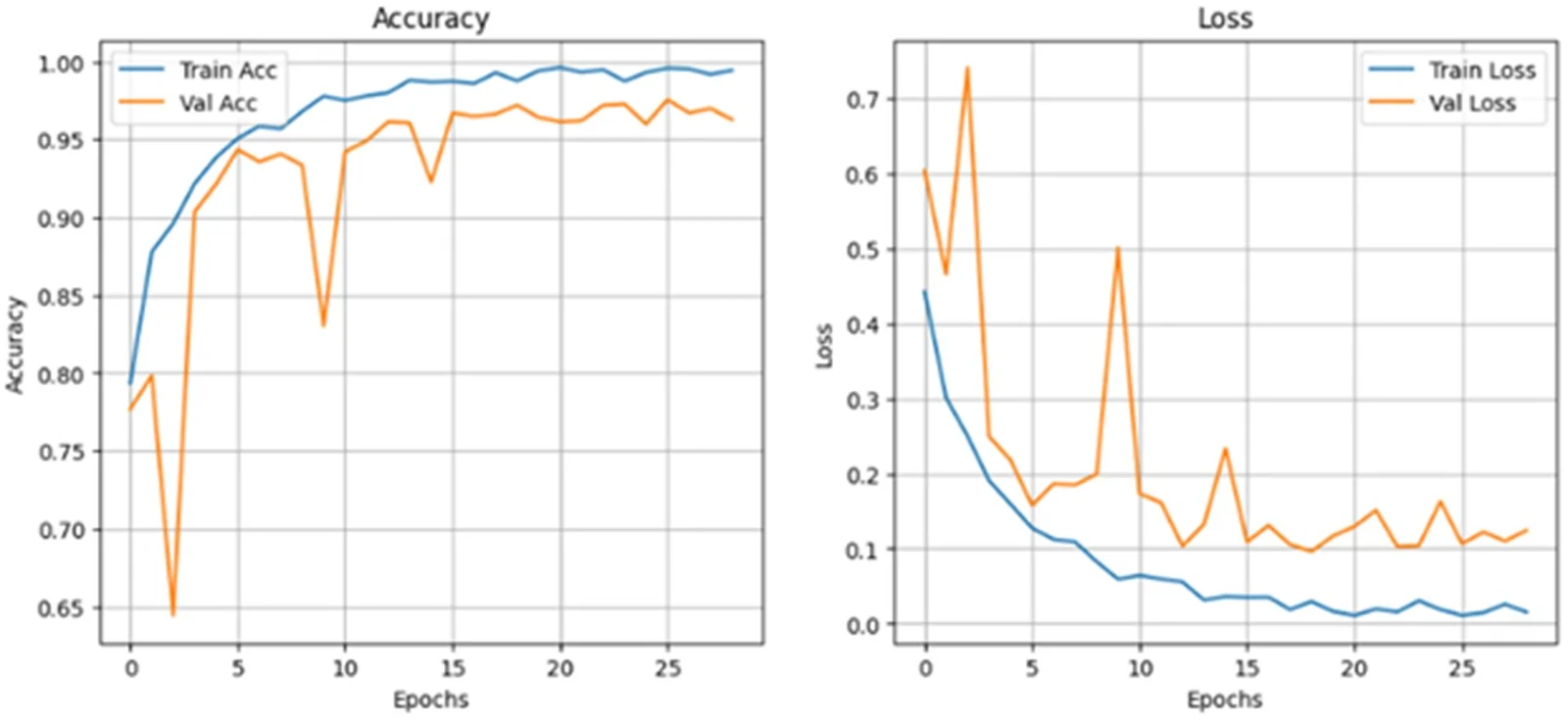
Accuracy and loss history plot.

This chart illustrates that the proposed model effectively captures patterns in the EEG seizure data while maintaining good generalization on the test set. The model achieves a AUC-ROC score of 0.9934. The training progress and performance history of the model are shown in Fig. 15.

The training history plots show that the MDCBG model learns quickly during the early epochs, with both training and validation accuracy rising sharply and stabilizing around 90%–93%, indicating strong and consistent performance. The validation accuracy closely follows the training accuracy, which suggests that the model is generalizing well without significant overfitting. The loss curves also show a steady decline for both training and validation sets, with validation loss fluctuating slightly due to noise in the data but overall following the same downward trend as training loss.

**Figure 16:**
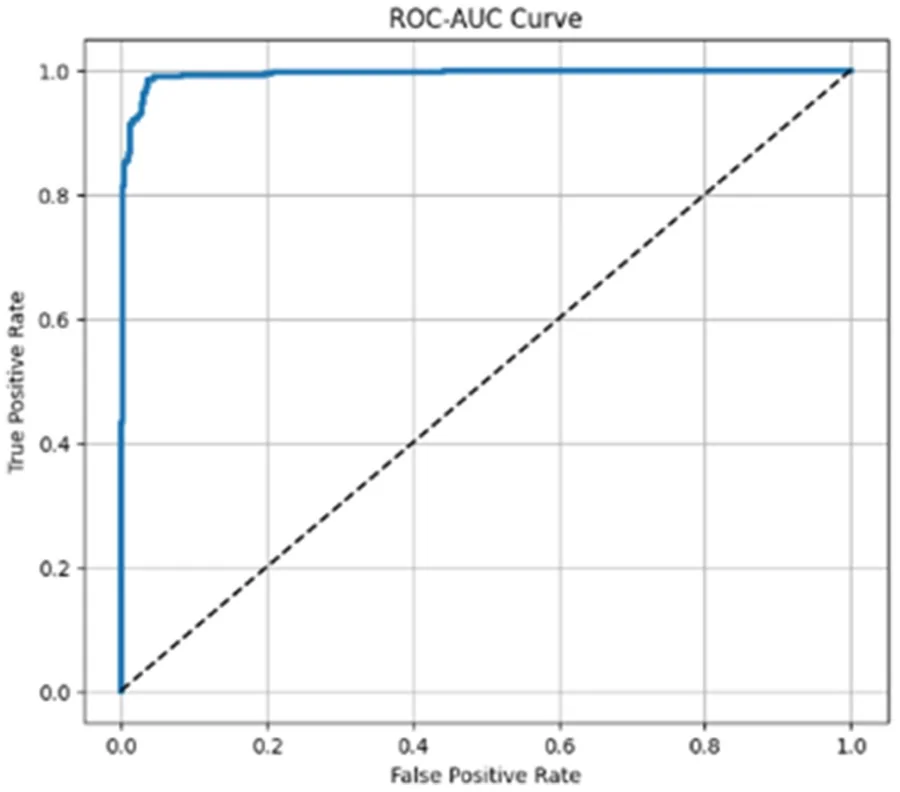
AUC-ROC plot.′

The certain spikes in validation loss and dips in validation accuracy occur mainly because multiple heterogeneous seizure types have been merged into a single “seizure” class. This aggregation increases intra-class variability, meaning that during some epochs the model encounters batches whose patterns differ significantly from what it has recently learned. As a result, the validation loss briefly increases (peaks) and accuracy drops before stabilizing again. This behavior indicates that the model is not overfitting but is instead adapting to complex class boundaries arising from the combination of diverse seizure manifestations. The recovery after each performance peak suggests strong generalization capability and learning robustness. With this adaptive behavior, the model’s potential to accurately identify a wide range of seizure types is significantly improved. The ROC AUC curve for this is given in Fig. 16.

Together, these plots indicate that the MDCBG model is training effectively, achieving high accuracy with stable convergence and no major signs of overfitting or underfitting. Importantly, after these fluctuations, both curves return to a stable trend:

- Training accuracy steadily improves and saturates near 1.0.
- Validation accuracy recovers and remains consistently high.
- Validation loss decreases overall and oscillates within a narrow range rather than diverging.

The training and validation plots demonstrate that the MDCBG model learns efficiently, with accuracy and loss curves converging smoothly, indicating stable optimization without noticeable overfitting or underfitting. As shown in Table 4, the MDCBG model consistently outperforms EEGNet and CTNet across all evaluation metrics, achieving higher precision, recall, and F1-scores for both classes, as well as the highest overall accuracy (0.97). This highlights its superior ability to correctly distinguish between seizure classes while maintaining balanced performance. Furthermore, the stratified cross-validation results in Table 5 confirm the robustness and generalization capability of the model, with a high mean test accuracy of 0.9696 ± 0.0101 and an excellent ROC-AUC of 0.9950 ± 0.0018, demonstrating consistent and reliable performance across different data splits.

**Table 4:** Models classification reports comparison.

Metric/class	MDCBG	EEGNet	CTNet
**Class 0 Precision**	0.99	0.88	0.80
**Class 0 Recall**	0.96	0.99	0.96
**Class 0 F1-score**	0.98	0.93	0.87
**Class 0 Support**	416	416	416
**Class 1 Precision**	0.96	0.99	0.94
**Class 1 Recall**	0.99	0.84	0.73
**Class 1 F1-score**	0.97	0.91	0.82
**Class 1 Support**	363	363	363
**Overall Accuracy**	0.97	0.92	0.85
**Macro Avg Precision**	0.97	0.93	0.87
**Macro Avg Recall**	0.98	0.92	0.84
**Macro Avg F1**	0.97	0.92	0.85
**Macro Avg Support**	779	779	779
**Weighted Avg Precision**	0.97	0.93	0.86
**Weighted Avg Recall**	0.97	0.92	0.85
**Weighted Avg F1**	0.97	0.92	0.85
**Weighted Avg Support**	779	779	779

**Table 5:** MDCBG model stratified cross validation performance.

Fold	Test Accuracy	ROC-AUC
**1**	0.9596	0.9931
**2**	0.9782	0.9967
**3**	0.9763	0.9970
**4**	0.9551	0.9926
**5**	0.9788	0.9957
**Mean ± SD**	0.9696 ± 0.0101	0.9950 ± 0.0018

**Table 6:** Model performance metrics comparison from ablation study.

Metric	CNN-only	BiGRU-only	No-Dropout MD-CNN-BiGRU
**Test accuracy**	0.9461	0.8485	0.9576
**Test ROC-AUC**	0.9892	0.9186	0.9919
**Precision (Weighted Avg)**	0.95	0.85	0.96
**Recall (Weighted Avg)**	0.95	0.85	0.96
**Total parameters**	3 342 273	484 097	3 966 401

#### Ablation study for proposed model (MDCBG)

For ablation study various models including CNN, BI-GRU, and no dropout MD-CNN-Bi-GRU are trained on seizure vs no-seizure data and metrics are compared as shown in Tables 6 and 7.

**Table 7:** Detailed class-level metrics (F1-Score).

Class	CNN-only	BiGRU-only	No-Dropout MD-CNN-BiGRU
**Class 0**	0.95	0.86	0.96
**Class 1**	0.94	0.84	0.95

The ablation research shows how each part of the suggested MD-CNN-BiGRU model contributes to the classification of seizures versus non-seizures. With a test accuracy of 0.9576, ROC-AUC of 0.9919, and weighted F1-score of 0.96, the No-Dropout MD-CNN-BiGRU outperforms the other two versions, highlighting the benefit of combining both spatial (CNN) and temporal (BiGRU) feature extraction.

The CNN-only model performs well, showing that spatial feature extraction alone can detect significant discriminative patterns in EEG data, with a test accuracy of 0.9461 and a ROC-AUC of 0.9892. The BiGRU-only model, on the other hand, does not perform better with a test accuracy of 0.8485 and ROC-AUC of 0.9186, indicating that temporal modeling alone is not adequate for the best seizure detection. Class-level F1-scores further support the hybrid MD-CNN-BiGRU model’s continuous improvement in prediction for both normal and seizure classes. In the end, these results validate the effectiveness of the proposed architecture, show that the selected MDCBG design provides better classification performance, and support the inclusion of both CNN and BiGRU blocks. These individual model-wise performances validate the effectiveness of the combined CNN and BiGRU blocks and demonstrate the significance of the proposed model compared to individual components.

#### Three-class performance comparison for models

##### EEGNet model performance

**Figure 17:**
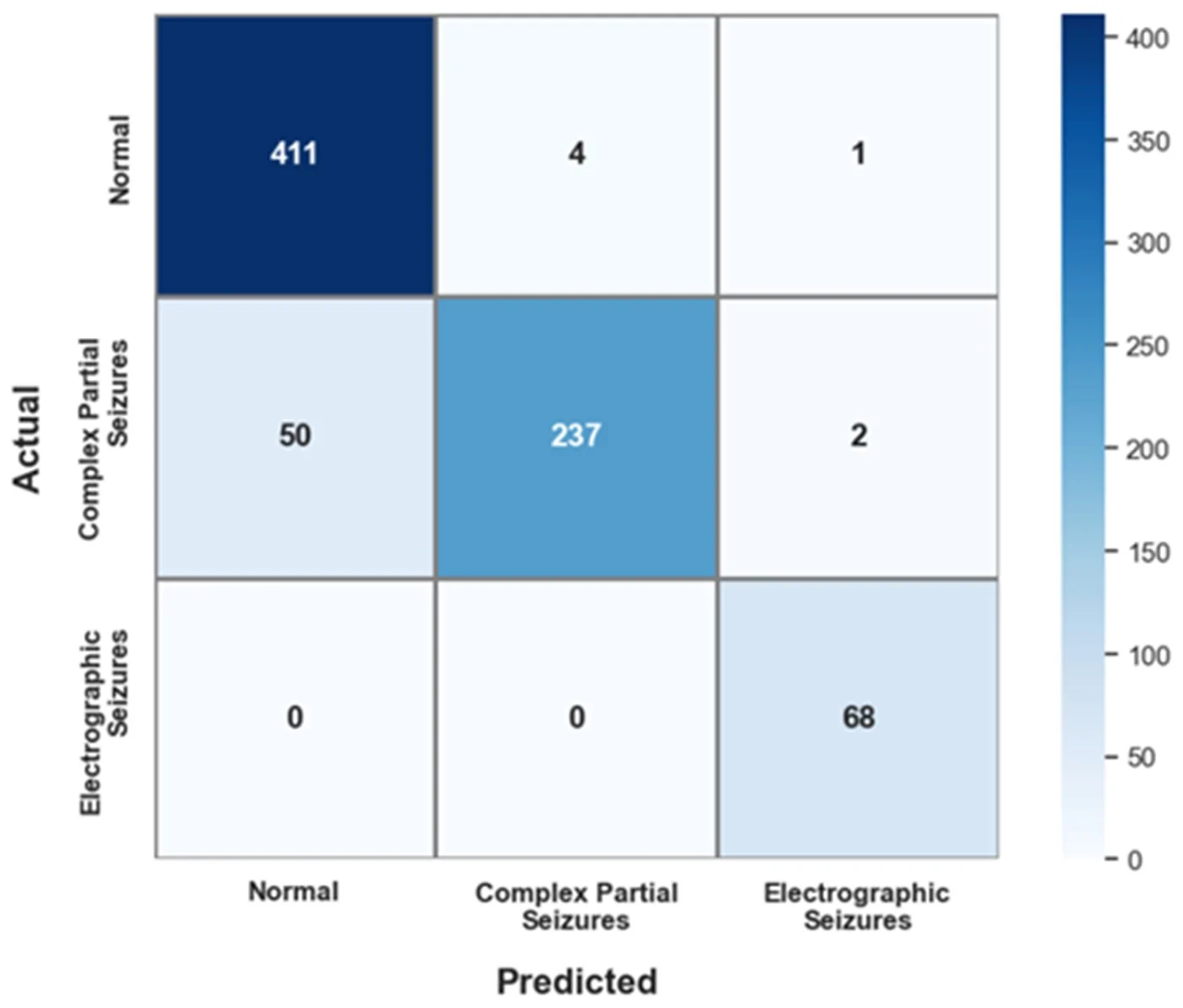
EEGNet confusion matrix plot.

The EEGNet obtained a training accuracy of 92.61% and a testing accuracy of 92.63%. The model EEGNet performs well on Classes 0 and 1, correctly predicting 411/416 and 237/289 samples respectively, showing strong accuracy for the majority classes. Class 2 also shows good performance with correct predictions of 68, without misclassifications as seen from Fig. 17.

**Figure 18:**
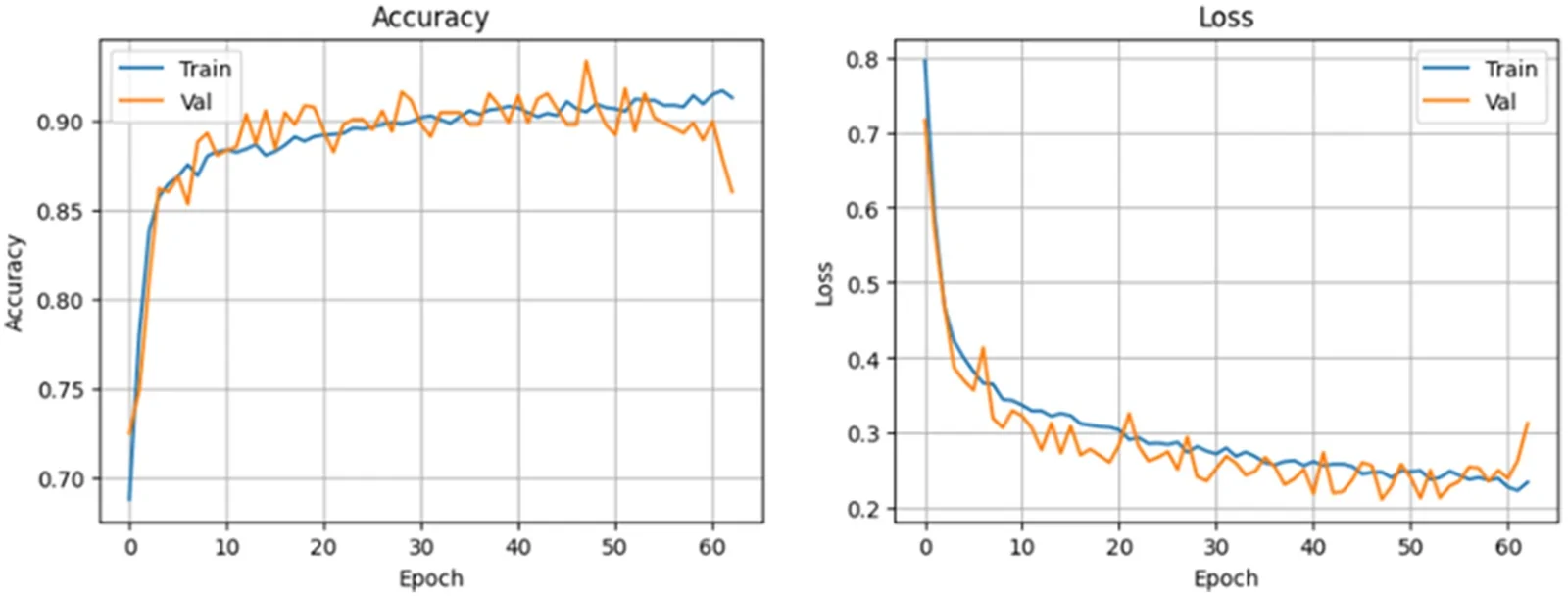
Model history plot for EEGNet.

As seen in Fig. 18, the training history graphs show a consistent convergence and good performance of the EEGNet model. The model is a bit affected by overfitting, as seen by the loss curve (left), evidenced by the growing gap where training loss decreases more than validation loss and training accuracy slightly outpaces validation accuracy in later epochs. Similarly, the accuracy curve (right) shows a steady upward trend, with training and validation accuracy gradually increasing and converging at about 90%. The model learns data patterns with some variations.

##### MDCBG performance

The proposed MDCBG model obtained a testing accuracy of 96.11% and a training accuracy of 99.18%.

**Figure 19:**
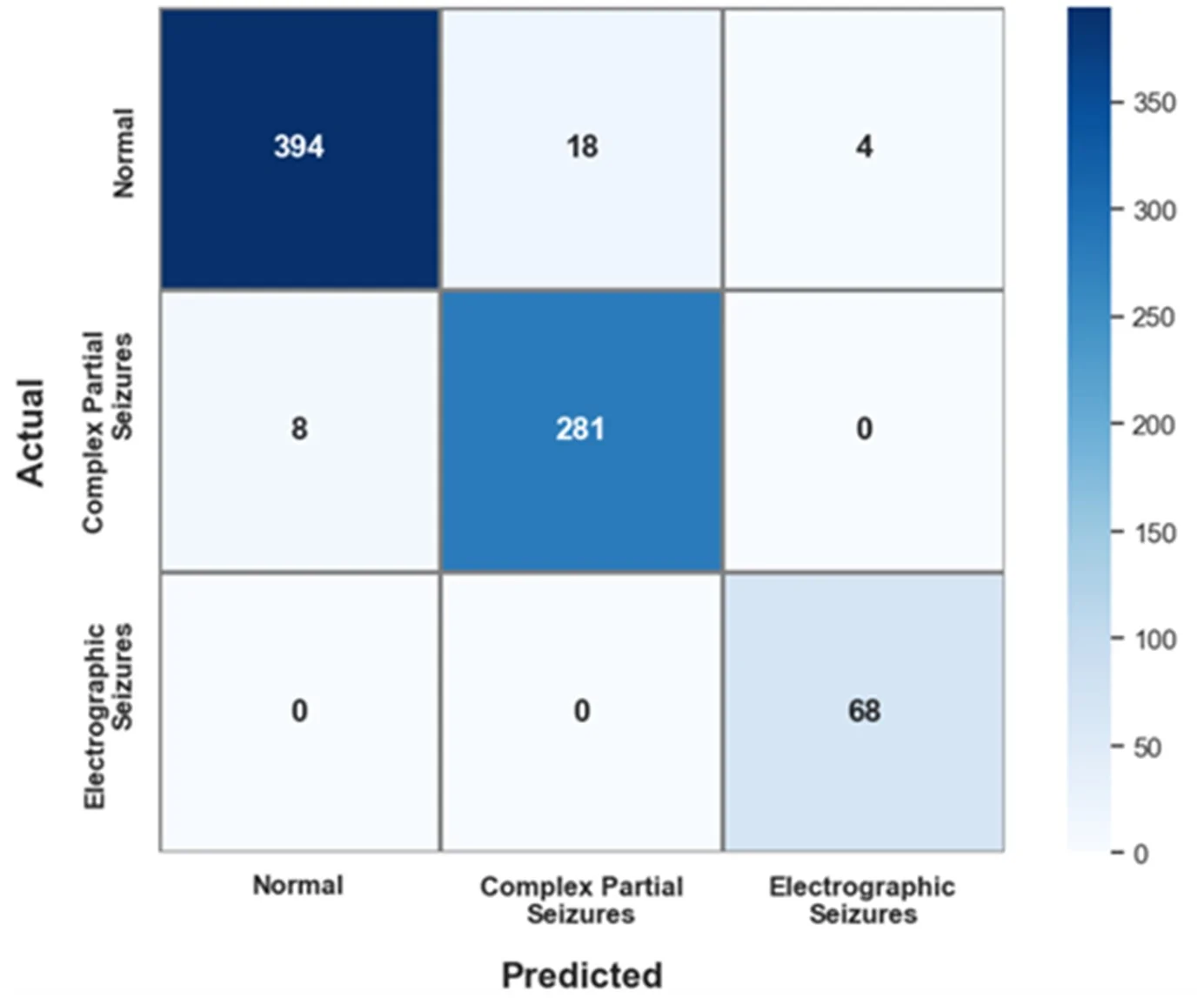
MDCBG model confusion matrix.

The model MDCBG performs very well on Classes 0 and 1, correctly predicting 394/416 and 281/289 samples respectively, showing strong accuracy for the majority classes. Class 2 also shows good performance with all correct predictions of 68 samples as seen in Fig. 19.

**Figure 20:**
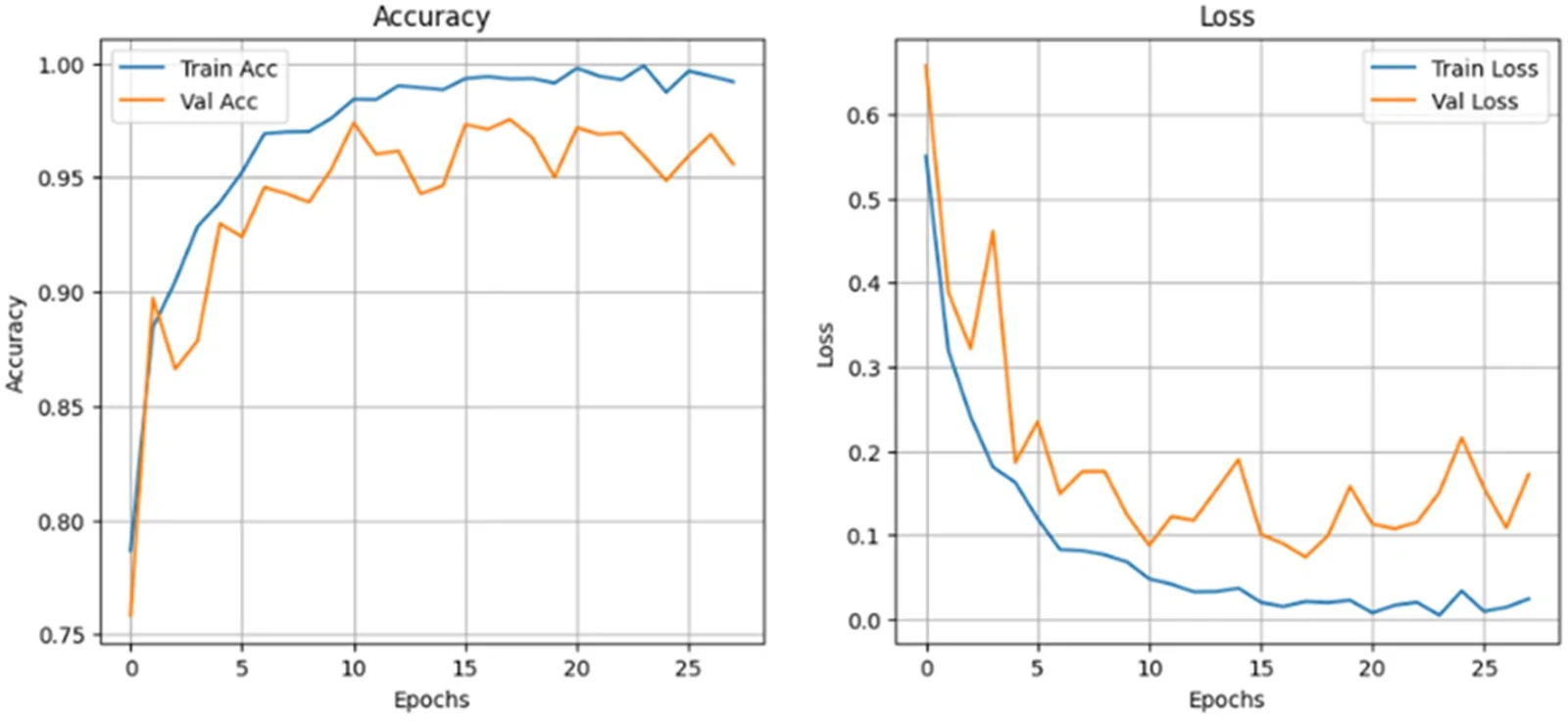
MDCBG history plot.

The MDCBG model learns rapidly in the early epochs, as seen by the training history plots in Fig. 20. Both training and validation accuracy rise substantially and stabilize around 90%–93%, demonstrating robust and reliable performance. The model appears to be generalizing well without severe overfitting, as evidenced by the validation accuracy roughly matching the training accuracy. Table 8 displays the cross-validation result. The MDCBG model plot indicates that it is training effectively, achieving high accuracy with stable convergence and no major signs of overfitting or underfitting. The model went through early stopping around epoch 28. The loss curves also show a steady decline for both training and validation sets, with validation loss fluctuating slightly due to noise in the data but overall following the same downward trend as training loss.

Table 8 shows that the model performed consistently well over all five folds, with accuracies of 0.9720, 0.9740, 0.9707, 0.9635, and 0.9550 for folds 1 through 5, respectively. The model’s performance is steady and insensitive to changes in the training and testing splits, as seen by its mean accuracy of 0.9671 and low standard deviation of 0.0070. This consistency reflects strong and dependable classification performance, indicating that the model has successfully acquired discriminative features from the EEG data and is expected to generalize well to unseen samples. The supplementary section lists additional models used for categorization along with their specific performance. Table 9 provides a detailed comparison of classification performance for each model.

**Table 8:** Model stratified cross validation result on three classes.

Fold	Accuracy
**Fold 1**	0.9720
**Fold 2**	0.9740
**Fold 3**	0.9707
**Fold 4**	0.9635
**Fold 5**	0.9550
**Mean ± SD**	0.9671 ± 0.0070

**Table 9:** Model wise classification report.

Model	Class	Precision	Recall	F1-Score	Support
**EEGNet**	0 (Normal)	0.89	0.99	0.94	416
	1 (CPS)	0.98	0.82	0.89	289
	2 (Electrographic)	0.96	1.00	0.98	68
	Accuracy	–	–	0.93	773
	Macro Avg	0.94	0.94	0.94	773
	Weighted Avg	0.93	0.93	0.92	773
**MDCBG**	0 (Normal)	0.98	0.95	0.96	416
	1 (CPS)	0.94	0.97	0.96	289
	2 (Electrographic)	0.94	1.00	0.97	68
	Accuracy	–	–	0.96	773
	Macro Avg	0.95	0.97	0.96	773
	Weighted Avg	0.96	0.96	0.96	773
**Bi-Directional GRU with Attention**	0 (Normal)	0.87	0.93	0.90	416
	1 (CPS)	0.88	0.82	0.85	289
	2 (Electrographic)	0.82	0.75	0.78	68
	Accuracy	–	–	0.87	773
	Macro Avg	0.86	0.83	0.84	773
	Weighted Avg	0.87	0.87	0.87	773
**Extra Trees Classifier**	0 (Normal)	0.86	0.98	0.92	416
	1 (CPS)	0.98	0.83	0.90	289
	2 (Electrographic)	0.92	0.71	0.80	68
	Accuracy	–	–	0.90	773
	Macro Avg	0.92	0.84	0.87	773
	Weighted Avg	0.91	0.90	0.90	773
**Random Forest**	0 (Normal)	0.85	0.97	0.91	416
	1 (CPS)	0.96	0.85	0.90	289
	2 (Electrographic)	0.81	0.50	0.62	68
	Accuracy	–	–	0.89	773
	Macro Avg	0.87	0.78	0.81	773
	Weighted Avg	0.89	0.89	0.88	773
**Bi-Directional LSTM GRU**	0 (Normal)	0.80	0.92	0.85	416
	1 (CPS)	0.85	0.73	0.79	289
	2 (Electrographic)	0.82	0.53	0.64	68
	Accuracy	–	–	0.82	773
	Macro Avg	0.82	0.73	0.76	773
	Weighted Avg	0.82	0.82	0.81	773
**XGBoost**	0 (Normal)	0.90′	0.97	0.93	416
	1 (CPS)	0.97	0.87	0.92	289
	2 (Electrographic)	0.90	0.84	0.87	68
	Accuracy	–	–	0.92	773
	Macro Avg	0.92	0.89	0.91	773
	Weighted Avg	0.93	0.92	0.92	773

**Table 10:** Model wise accuracies obtained for types of seizure prediction.

Model	Testing accuracy	AUC-ROC score
**Bi-Directional GRU with Attention**	87%	0.9554
**Extra Trees Classifier**	90.29%	0.9587
**Random Forest**	88.61%	0.9604
**Bi-Directional LSTM GRU**	82%	0.9268
**XGBoost**	92.23%	0.98058
**EEGNet**	92.61%	0.9846
**Proposed MDCBG**	96.11%	0.99614

The classification performance of various models for seizures is displayed in Table 9. Two of the deep learning models, Bi-Directional GRU with Attention and Bi-Directional LSTM GRU, showed good but different classification performance, with testing accuracies of 87% and 82% and AUC-ROC scores of 0.9554 and 0.9268, respectively. Conventional ensemble models like Extra Trees and Random Forest performed slightly better, with accuracies of 90.29% and 88.61% and AUC-ROC scores above 0.95. Gradient boosting with XGBoost improved performance even more, achieving 92.23% accuracy and 0.98058 AUC-ROC. EEGNet demonstrated good generalization with an accuracy of 92.61% and an AUC-ROC of 0.9846. With the greatest testing accuracy of 96.11% and an AUC-ROC of 0.99614, the proposed MDCBG model demonstrated exceptional predictive performance and robustness, outperforming all other models in this classification challenge.

### SHAP analysis results

When using EEGNet or similar neural networks, the EEG data is typically reshaped (e.g. into 2D or 3D tensors) to fit the network’s input requirements, which makes plotting SHAP values for the original EEG channels directly more challenging. With EEGNet alone, SHAP can attribute model predictions to the raw EEG channels and their temporal patterns, since the network operates on channel-wise time series data. However, because of the data reshaping and feature transformations inside the network, the SHAP explanations correspond to processed features rather than the original EEG channels, making direct interpretation on the raw signals more difficult.

**Figure 21:**
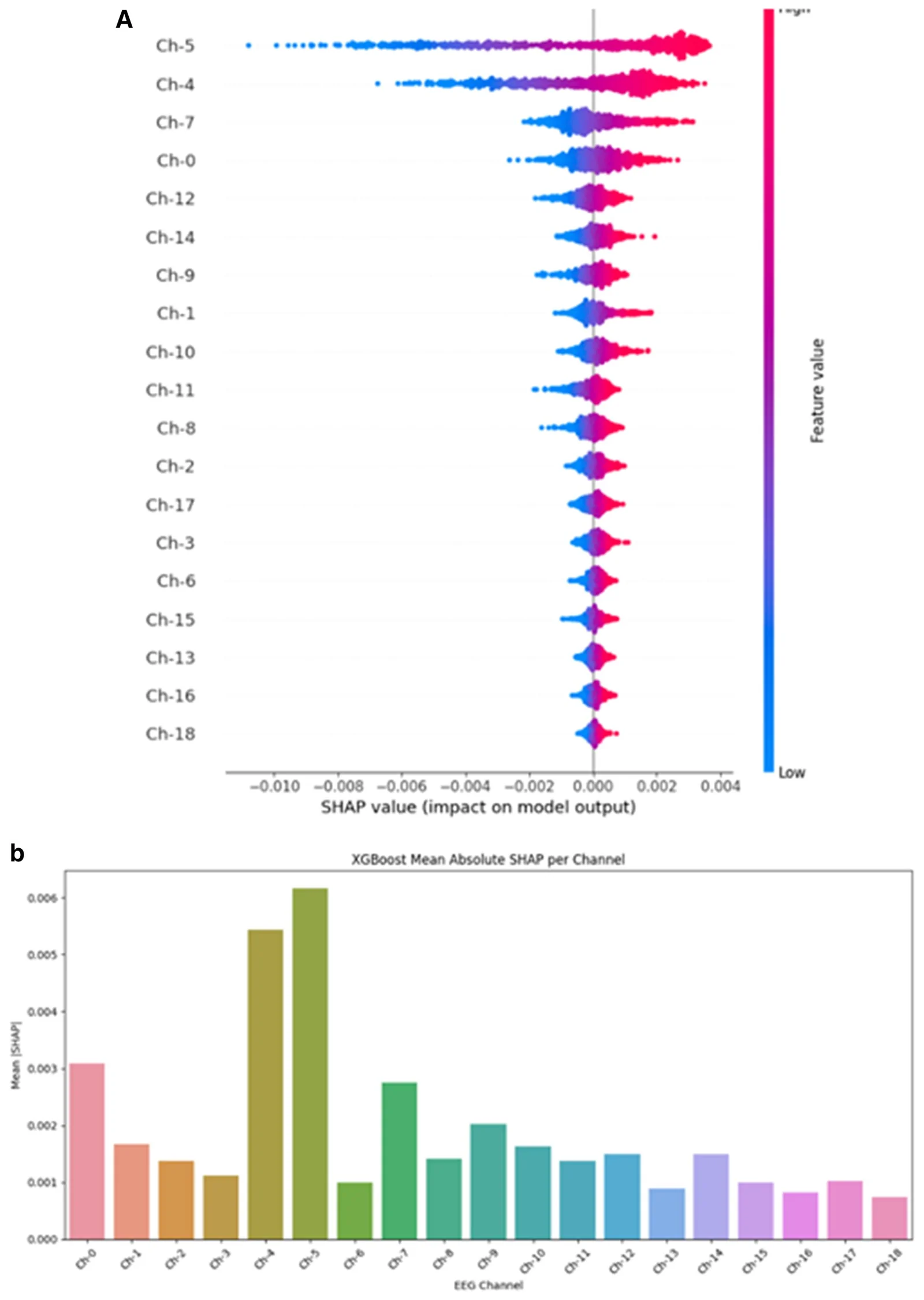
(a) SHAP plot for model. (b) SHAP bar plot for model.

In EEG analysis, each channel records a time series of electrical activity, resulting in multiple time points per channel. When computing SHAP values for models like XGBoost trained on flattened EEG data, each timepoint is treated as a separate feature, producing an extremely high-dimensional SHAP output as seen in Fig. 21a and b. Aggregating SHAP values over timepoints collapses this temporal dimension, producing a single SHAP value per channel for each sample. This aggregation is significant because it allows us to visualize the overall contribution of each EEG channel to the model’s prediction without being overwhelmed by thousands of individual timepoint features. It aligns the interpretability of SHAP with the physiological structure of EEG data, enabling easier comparison across channels and clearer insights into which brain regions are most influential for the classification task. By doing so, we preserve the model’s explanation at the channel level, which is more meaningful and interpretable for neuroscientific and clinical analysis. The Table 10 shows detailed performance for each models.

Interestingly, Channel 5 (Ch-5) has the highest mean feature importance, indicating that it contains the most discriminative information for the task. This could indicate that there is a large amount of brain activity associated with the condition under study. While some channels contribute less and cluster closer to zero, others, like Ch-4 and Ch-0, also have very significant relevance. By identifying important brain regions for additional research or focused electrode installation, these insights enhance the interpretability of the model and its potential for clinical use. The figure emphasizes the importance of feature selection in EEG-based machine learning by demonstrating that not all channels are equally useful.

The bar chart in Fig. 21b displays the average feature importance of different EEG channels (Ch-0 to Ch-18) as computed by the XGBoost model. Each bar represents how much a particular channel contributes to the model’s predictions on average across timepoints. Among all channels, Ch-5 stands out significantly, showing the highest mean feature importance by a large margin. This indicates that Ch-5 carries the most critical information for distinguishing between classes in the seizure detection task, and SHAP values also quantify how strongly each feature influences model predictions. Therefore, this confirms that Ch-5 has the greatest impact on model performance, suggesting it may capture key patterns or signals relevant to epileptic activity in the EEG data.

### IoT-based alert system

**Figure 22:**
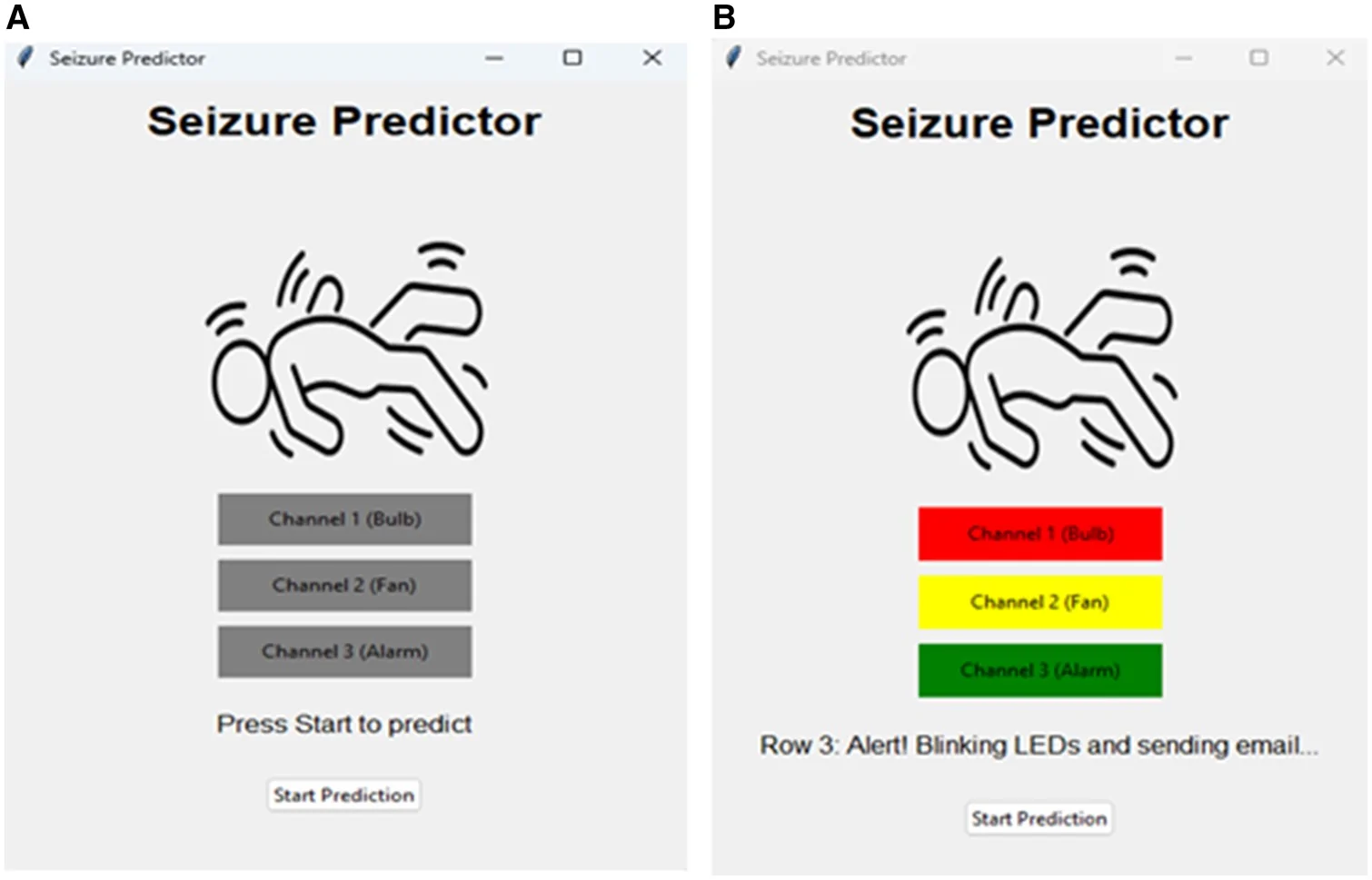
(a) Application for automation simulation. (b) Appliances triggered simulated for seizure prediction

**Figure 23:**
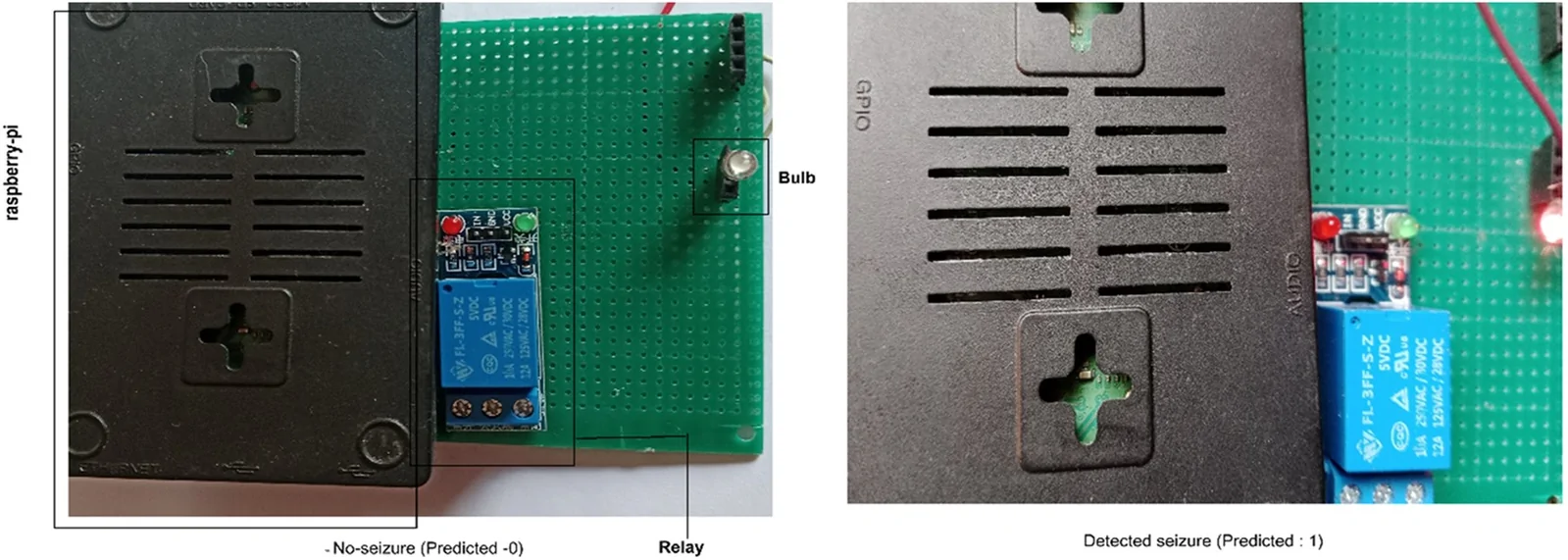
System configuration for seizure prediction

Figure 22a and b shows the tkinter-based interface for the BCI program that connects to IoT systems, initiates when a seizure is detected, and sends emails. Figure 23 shows the load that is triggered when a seizure is predicted via the model deployed in the Raspberry Pi microcomputer system. This works well for giving victims early assistance. In order to categorize seizures and replicate the scenarios, fresh test set data is fed into the model. For improved performance and dependable prediction in IoT systems, the data can be collected via an EEG headset, stored on the cloud, and used to train model that can be used for inference.

**Figure 24:**
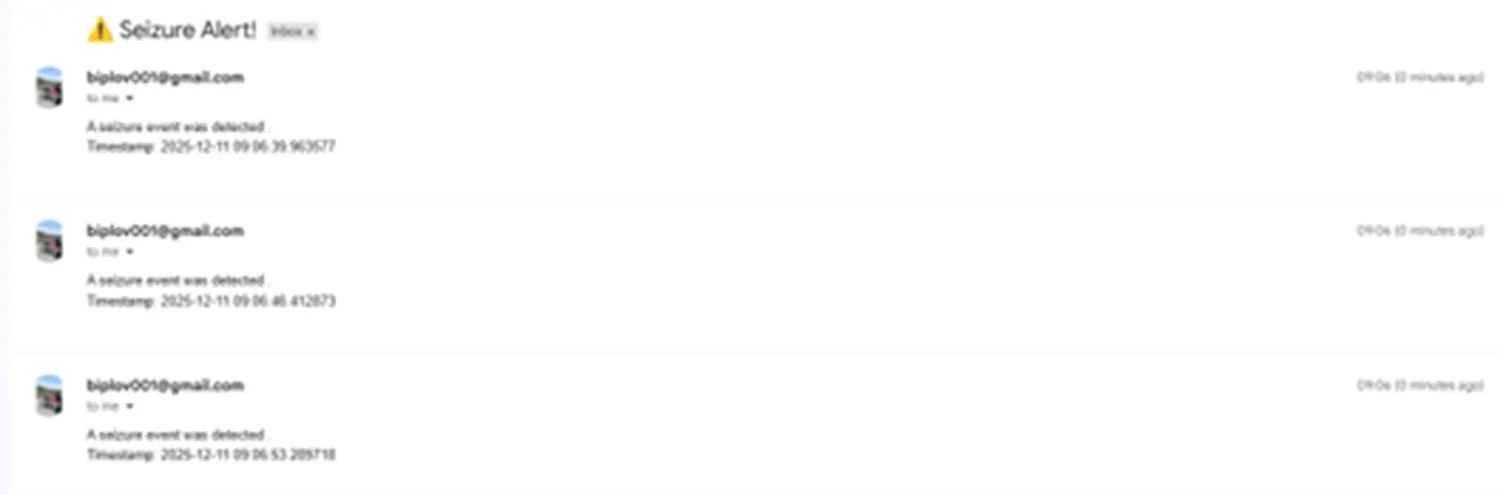
Seizure email alert via IoT SMTP

Figure 23 shows the seizure prediction-based trigger of appliances in the case when a seizure is predicted by the model. This system ensures that the email reaches the caretaker in the case of emergency. This simulation is a proof-of-concept; when a victim puts on a headset and a seizure is predicted with the help of the trained model, an alert notification can be sent. The email sent through SMTP is shown in Fig. 24. These results indicate that the MDCBG model successfully learned to distinguish between the different classes, achieving strong and reliable performance with minimal overfitting concerns. The hyperparameters utilized for different models are displayed in Tables S1–S4 in the Supplementary Appendix section. Table S5 lists the different system software components, and Table S6 displays the hardware components and Table S7 displays the model deployment fasibility results in the Supplementary Appendix.

With this system as a proof-of-concept early warning can be triggered, and early alert notification can be sent via IoT on email. This system automates appliances by using microcomputer-based processing of the EEG signals from the patient, allowing for the automation of room conditions—such as turning on a bulb, fan, or similar device—when the model predicts the seizure through relay-based triggering. In summary, this system acts as feedback for making an IoMT (Internet of Medical Things) home automation for seizure victim aid and managing favorable conditions to provide a relief aspect to the victim. Thus, patients can be aided easily with the help of the early warning and notification system along with the control mechanism for various devices in the home environment.

### Limitations and future works

Therefore, the suggested method shows a substantial improvement in EEG signal categorization for identifying and categorizing different kinds of seizures. It is important for precise prediction as well as for determining the most important characteristics and channels that impact model performance, which ultimately directs the choice of the best model to improve. Even if the current results outperform baseline models, there is still room for improvement. Cross-validation has been used to lower the risk of overfitting and guarantee that the model performs robustly and generalizes effectively across unknown data. Furthermore, AUC-ROC (Area Under the Receiver Operating Characteristic curve) evaluation offers a trustworthy metric for evaluating the model’s capacity to distinguish between seizure and non-seizure events, providing a more thorough understanding of classification performance than just accuracy. Because of the Raspberry Pi’s slow processing speed, the deployed model experienced computation lag; hence, a higher version of it may be useful.

The lack of training data for class 3 (videographic seizures) in the dataset prevented the model from being trained and evaluated, which is a limitation of the current study. To increase the model’s resilience and capacity for generalization, future research should concentrate on gathering and integrating a bigger dataset for this class. To improve the robustness of the model, future research should concentrate on incorporating a larger dataset for this class. Due to the combination of many seizure types into a single seizure class, the binary models that distinguished between seizure and non-seizure states showed slight learning challenges.

Although the proposed models demonstrate strong performance in terms of accuracy (96%–97%), F1-score, and macro-averaged ROC–AUC, these results are obtained under offline experimental conditions. Therefore, this system acts a proof-of-concept for future developments. Real-time seizure detection is influenced by several external factors, including signal acquisition latency, hardware constraints, noise variability, and computational overhead, none of which were evaluated in the current setup. Aiding victims immediately with this BCI approach offers a significant step for biomedical enhancements. This behavior is evident in the loss curves. Nevertheless, these models achieved higher accuracy, F1-scores, and macro-averaged ROC–AUC values, while also demonstrating reduced susceptibility to overfitting. Although the developed models achieve 95%–97% accuracy, this level is still not fully reliable as a proof-of-concept deployment in an automation ecosystem. Furthermore, while the study demonstrates the potential of an IoMT-based approach to aid patients, real-time evaluation could not be performed due to the lack of EEG headsets and advanced hardware. Future studies should prioritize rigorous prototyping and real-time testing to validate practical deployment utilizing suitable headset-based new data acquisition.

## Conclusion

To conclude, a hybrid deep learning model like MDCBG has a higher potential to gather greater performance for EEG results with approximately 97% accuracy for seizure detection. The models trained on the dataset show that prior to various epileptic conditions, early warning can be triggered using ML-based detection via victim EEG data. The EEG preprocessing and normalization applied allowed the hybrid deep learning models to perform and learn patterns very well. This approach can be useful for deployment in real-time prototyping for IoMT edge and medical device development. With this approach, future intelligent biomedical systems based on EEG can be enhanced.

## Supplementary Material

bpag010_Supplementary_Data

## Data and codes availability

Data is publicly available at: Nasreddine, W., 2021. Epileptic EEG dataset. Mendeley Data, V1. https://doi.org/10.17632/5pc2j46cbc.1. Further the model development codes and files are available at github repository: Biplov01/EEG_MDCBG_Hybrid.

## Declaration of LLM’s use

The authors used LLM tools for minimizing grammatical errors and writing.
