# Supplementary material for: A multi-dimensional CNN-Bi-GRU for IoT-based brain–computer interface in early epileptic seizure detection
Source: Biol Methods Protoc. 2026 Feb 17;11(1):bpag010. doi: 10.1093/biomethods/bpag010 (PMC13049591; doi:10.1093/biomethods/bpag010)
Supplement: bpag010_Supplementary_Data [file bpag010_supplementary_data.zip › supplementary_cleaned_final_2_15.docx]

**Appendix:**

1. **Performance on 3 classes for various models**
2. Bi-Directional GRU with attention method:

The confusion matrix for Bi-Directional GRU with attention approach model obtained a training Accuracy of 92% and testing accuracy of 87%.


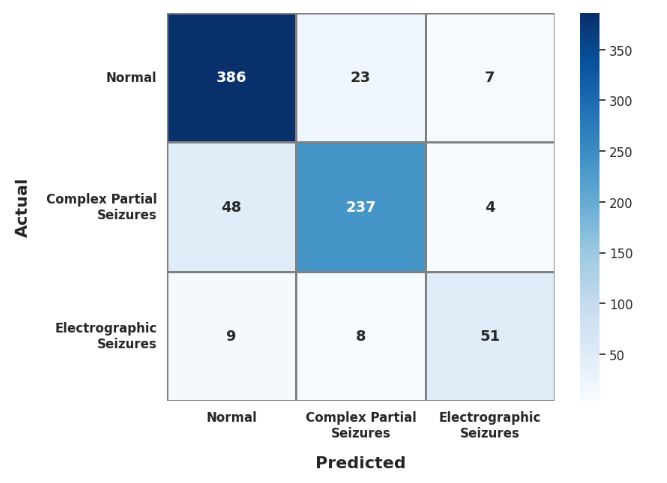


Fig 1. Confusion matrix for Bi-directional GRU with attention

The confusion matrix plot in figure 1 is displayed as seen in figure in which for class ‘0’ 386 instances or testing feature set were correctly classified. Similarly, for class ‘1’ 237 instances were correctly classified as class ‘1’ and ‘51 instances for class ‘2’.

~~
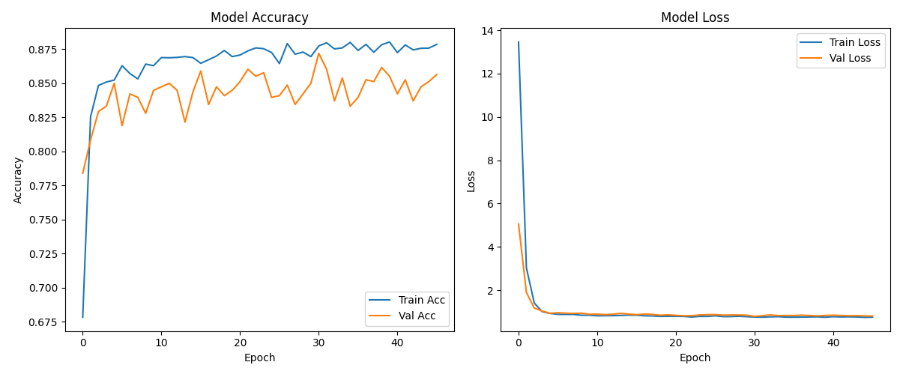
~~

Fig 2. Training and loss history plot

The model shown good development over epochs with improvements as shown in figure 2. The training history plots illustrate how the model learns over approximately 45 epochs. In the left plot showing accuracy, both training and validation accuracy rise sharply in the initial epochs, indicating that the model quickly captures key patterns in the data. The training accuracy stabilizes around 0.88–0.90, while the validation accuracy plateaus slightly lower, around 0.83–0.85, suggesting that the model is fitting well but retains a modest gap between training and validation performance. In the right plot, depicting loss curves, both training and validation loss decrease steeply at first and then flatten out, maintaining similar levels from about epoch 10 onward as shown in figure 2.

1. **Extra Trees classifier results:**

The model Extra Trees classifier obtained a training Accuracy: 96.7% and testing Accuracy: 90.29% and confusion matrix is depicted in figure 3.


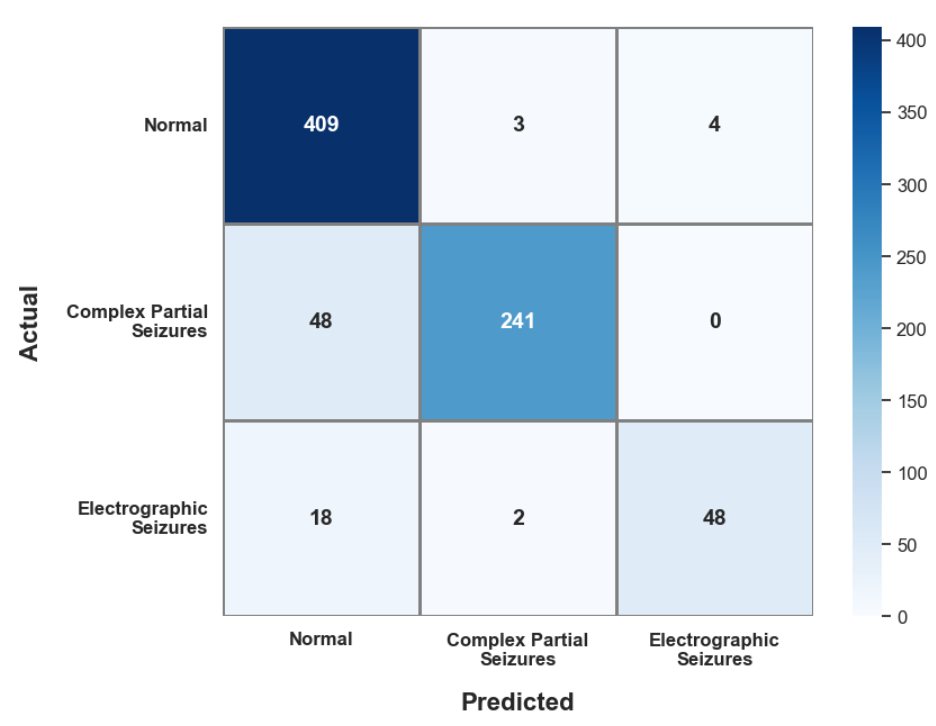


Fig 3: Confusion matrix plot for Extra Trees Classifier model

1. **Random Forest model**

The model obtained a training accuracy of 100% as well as testing accuracy of: 88.61%. The confusion matrix plot is displayed as seen in figure ~~4~~ in which 404 instances or testing feature set were correctly classified for class ‘0’ similarly, for class ‘1’ 247 instances were correctly classified as class ‘1’ and ‘34’ instances for class ‘2’ as shown in figure 4.


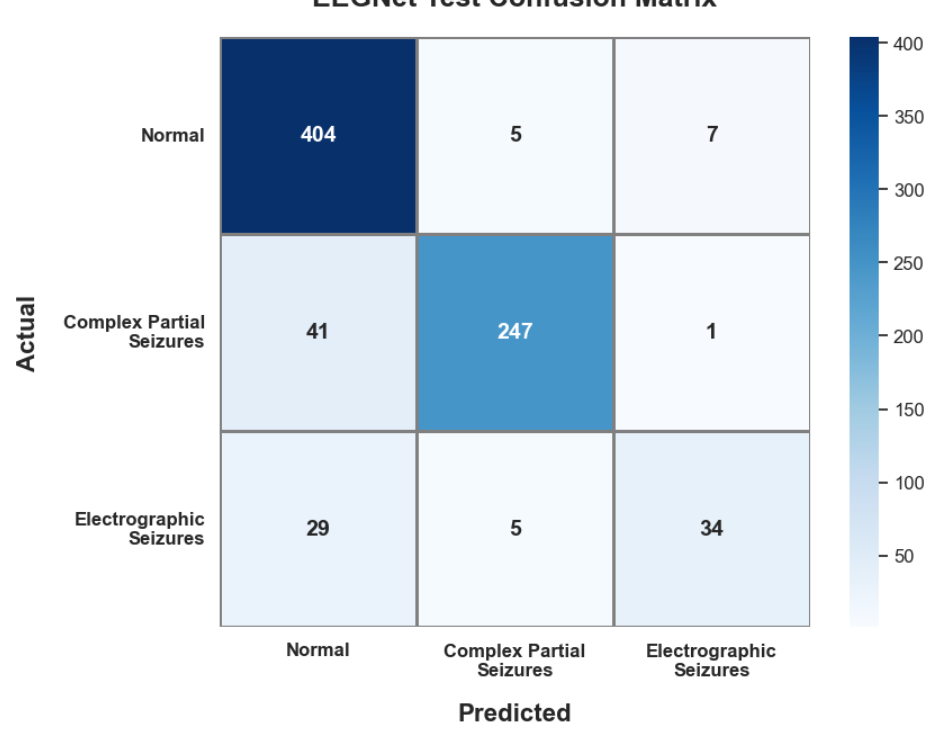


Fig 4: Random forest model confusion matrix plot

1. **Bi-Directional LSTM GRU:**

The model obtained a training Accuracy 88% including a testing accuracy of 82%. The confusion matrix plot is displayed as seen in figure in which for class ‘0’ 382 instances or testing feature set were correctly classified similarly, for class ‘1’ 212 instances were correctly classified as class ‘1’ and ‘36 instances for class ‘2’ as shown in figure 5.


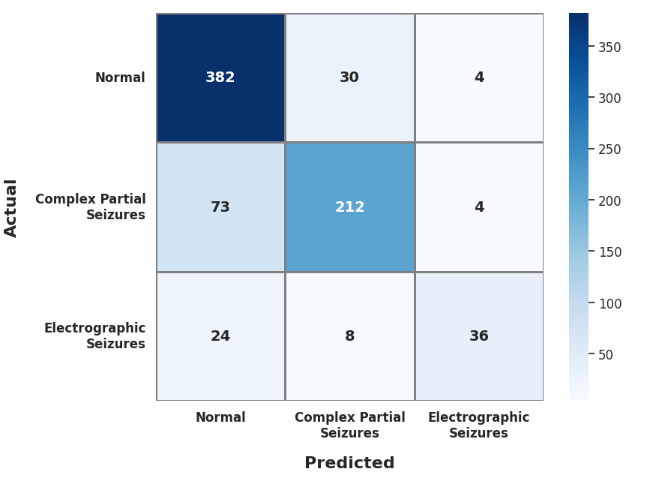


Fig 5: Confusion matrix plot for Bi-directional LSTM-GRU model

~~
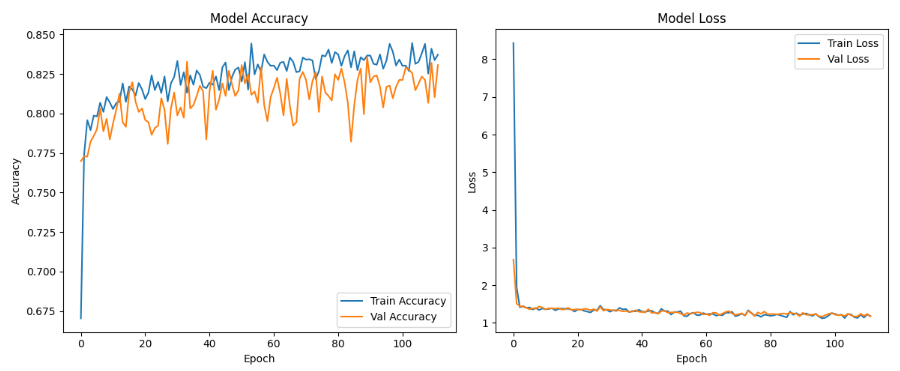
~~

Fig 6: Bi-Directional LSTM GRU training and loss history

The model as shown in figure 6 show good development over epochs with improvements. The training history plots illustrate how the model learns over approximately 110 epochs. In the left plot showing accuracy, both training and validation accuracy rise sharply in the initial epochs, indicating that the model quickly captures key patterns in the data. The training accuracy stabilizes around 0.85-0.87, while the validation accuracy plateaus slightly lower, around 0.80-0.83, suggesting that the model is fitting well but retains a modest gap between training and validation performance. In the right plot, depicting loss curves, both training and validation loss decrease steeply at first and then flatten out, maintaining similar levels from about epoch 10 onward. The close alignment between training and validation loss throughout training suggests that the model generalizes reasonably well and does not suffer from significant overfitting or underfitting. Overall, these plots indicate a stable and effective training process, achieving good performance while maintaining consistency between training and validation results.

1. **XGBoost model:**

The model obtained an accuracy of 100% on training set and Testing Accuracy: 92.23% accuracy on testing set. The confusion matrix plot in figure 7 is displayed as seen in figure in which 405 instances or testing feature set were correctly classified for class ‘0’ similarly, for class ‘1’ 251 instances were correctly classified as class ‘1’ and ‘57’ instances for class ‘2’.


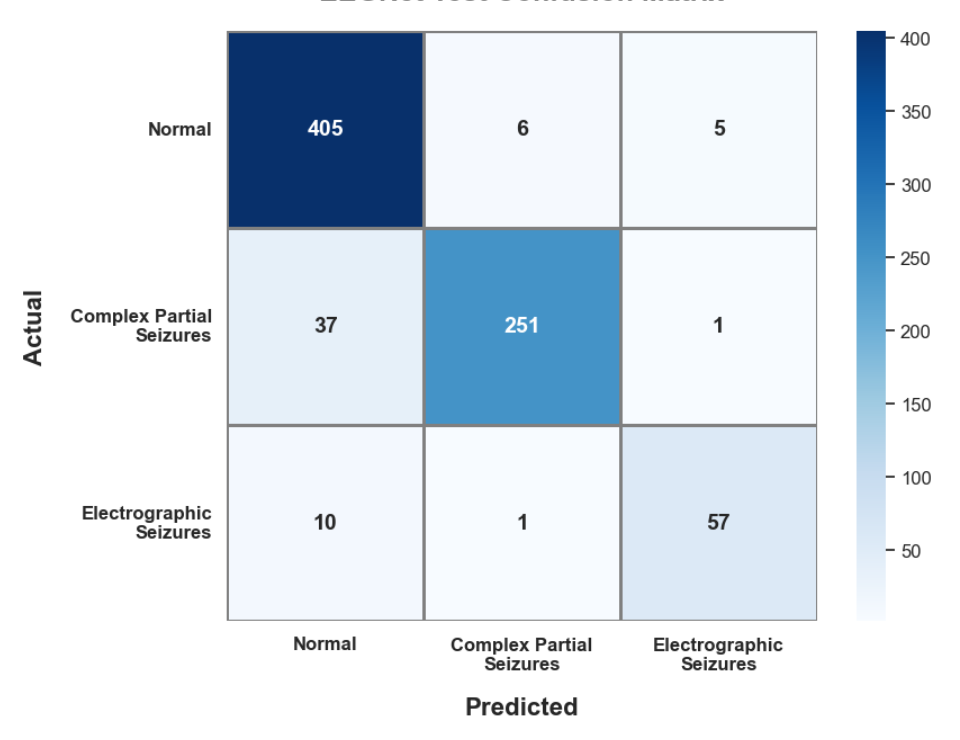


. Fig 7: Confusion matrix plot for XGBoost

1. **Hyperparameters for various models**

Table 1 presents the hyperparameter settings for the tree-based and traditional machine learning models used in this study. Table 2 summarizes the hyperparameters of the Bi-LSTM-GRU model, while Table 3 details the configuration of the bi-directional GRU model with attention. Finally, Table 4 lists the hyperparameters of the proposed MDCBG (Multi-Dimensional CNN Bi-GRU) model. Together, these tables provide a complete overview of the parameter choices and architectural settings used for each model evaluated in this work.

**Table 1:** Training Hyperparameters for traditional models

| Model | Hyperparameters |
| --- | --- |
| XGBoost | max_depth = 50n_estimators = 1000eval_metric = 'mlogloss'random_state = 42 |
| Random Forest | n_estimators = 100class_weight = 'balanced'random_state = 42 |
| Extra Trees | n_estimators = 300max_depth = 30min_samples_split = 5min_samples_leaf = 2max_features = 'sqrt'bootstrap = Trueclass_weight = 'balanced'random_state = 42n_jobs = -1 |

**Table 2:** BI-LSTM-GRU hyperparameters

| **Category** | **Hyperparameter** | **Value** |
| --- | --- | --- |
| **Data Handling** | Dataset | Epilepsy EEG (Kaggle) |
|  | Class excluded | Class 3 (Video-detected seizures) |
|  | Feature flattening | EEG reshaped to 2D |
|  | Normalization | StandardScaler |
|  | RNN input shape | (samples, 1, features) |
| **Model Architecture** | Model type | Sequential |
|  | Recurrent layers | Bidirectional LSTM → Bidirectional GRU |
|  | LSTM units | 128 |
|  | LSTM return sequences | True |
|  | GRU units | 128 |
|  | Output layer | Dense |
|  | Output neurons | number of classes (after exclusion) |
|  | Output activation | Softmax |
| **Regularization** | L2 penalty | 0.01 |
|  | Dropout rate | 0.2 |
| **Training Setup** | Optimizer | Adam |
|  | Loss function | Sparse Categorical Cross-Entropy |
|  | Batch size | 128 |
|  | Maximum epochs | 200 |
|  | Validation split | 0.2 |
|  | Early stopping | Enabled |
|  | Early stopping monitor | val_loss |
|  | Early stopping patience | 15 |
|  | Restore best weights | True |
| **Evaluation Metrics** | Primary metric | Accuracy |
|  | Additional metrics | Confusion Matrix, Classification Report |
|  | ROC-AUC | Macro & Micro (One-vs-Rest) |

Table 3: Bi-GRU with attention hyperparameters:

| **Category** | **Hyperparameter** | **Value** |
| --- | --- | --- |
| **Data Handling** | Dataset | Epilepsy EEG (Kaggle) |
|  | Class removed | Class 3 (Video-detected seizures) |
|  | Feature flattening | EEG reshaped to 2D |
|  | Normalization | StandardScaler |
|  | RNN input shape | (samples, 1, features) |
| **Model Architecture** | Model type | Sequential |
|  | Recurrent layer | Bidirectional GRU |
|  | GRU units | 256 |
|  | Return sequences | True |
|  | Attention mechanism | Custom trainable attention layer |
|  | Output layer | Dense |
|  | Output neurons | number of classes (after exclusion) |
|  | Output activation | Softmax |
| **Attention Layer** | Weight matrix shape | (hidden_units, 1) |
|  | Bias | Trainable scalar |
|  | Attention type | Additive (softmax-based) |
| **Regularization** | L2 regularization | 0.01 |
|  | Dropout rate | 0.2 |
| **Training Setup** | Optimizer | Adam |
|  | Learning rate | 5 × 10⁻⁴ |
|  | Loss function | Sparse Categorical Cross-Entropy |
|  | Batch size | 128 |
|  | Maximum epochs | 500 |
|  | Validation strategy | External (test set) |
|  | Early stopping | Enabled |
|  | Early stopping monitor | val_loss |
|  | Early stopping patience | 15 |
|  | Restore best weights | True |
| **Evaluation Metrics** | Primary metric | Accuracy |
|  | Additional metrics | Confusion Matrix, Classification Report |
|  | ROC-AUC | Macro & Micro (One-vs-Rest) |

**Table 4:** Model MDCBG hyperparameters

| **Component** | **Hyperparameter** | **Value** |
| --- | --- | --- |
| **Model Input** | Channels | 1 |
|  | Depth (EEG channels) | 19 |
|  | Time points | 500 |
|  | Input Shape | (19, 500, 1, 1) |
| **Conv3D Block 1** | Filters | 32 |
|  | Kernel size | (3,3,3) |
|  | Stride | (1,2,2) |
|  | Activation | ReLU |
|  | Padding | Same |
| **MaxPool Block 1** | Pool size | (1,2,2) |
|  | Stride | (1,2,2) |
|  | Padding | Same |
| **Conv3D Block 2** | Filters | 64 |
|  | Kernel size | (3,3,3) |
|  | Activation | ReLU |
|  | Padding | Same |
| **MaxPool Block 2** | Pool size | (2,2,2) |
|  | Stride | (2,2,2) |
|  | Padding | Same |
| **Conv3D Block 3** | Filters | 128 + 128 |
|  | Kernel size | (3,3,3) |
|  | Activation | ReLU |
|  | Padding | Same |
| **MaxPool Block 3** | Pool size | (2,2,2) |
|  | Stride | (2,2,2) |
|  | Padding | Same |
| **Conv3D Block 4** | Filters | 128 + 128 |
|  | Kernel size | (3,3,3) |
|  | Activation | ReLU |
|  | Padding | Same |
| **MaxPool Block 4** | Pool size | (2,2,2) |
|  | Stride | (2,2,2) |
|  | Padding | Same |
| **Batch Normalization** | - | Yes |
| **BiGRU** | Units | 128 |
|  | Return sequences | False |
|  | Bidirectional | Yes |
| **Dropout** | Rate | 0.5 |
| **Dense Output** | Units | 1 |
|  | Activation | Sigmoid |
| **Optimizer** | Adam | Yes |
|  | Learning Rate | 1e-3 |
| **Loss Function** | Binary Crossentropy | Yes |
| **Metrics** | Accuracy | Yes |
| **Training** | Epochs | 200 |
|  | Batch size | 32 |
|  | Validation split | 0.2 |
| **Early Stopping** | Monitor | val_loss |
|  | Patience | 15 |
|  | Restore best weights | True |
| **Prediction Threshold** | 0.5 | Yes |

1. **Software and Hardware Configuration:**

Model development trainings were conducted on Kaggle using the default runtime environment. The Python version was 3.11.13, with TensorFlow 2.18.0, NumPy 1.26.4, and Scikit-learn 1.2.2. GPU acceleration was enabled using an NVIDIA Tesla P500 GPU. The operating system was Linux 6.6.105+.

Random seeds: All experiments used a fixed random seed of 42 for reproducibility in traditional models and training. Table 5 and table 6 show various software and hardware components with versions. Table 7 finally reports various metrics for model deployment on Raspberry Pi.

Table 5: system software components & their versions

| **Component** | **Version / Detail** |
| --- | --- |
| Python | 3.11.13 |
| TensorFlow | 2.18.0 |
| NumPy | 1.26.4 |
| Scikit-learn | 1.2.2 |
| GPU Accelerator | NVIDIA Tesla P500 |
| OS | Linux 6.6.105+ |
| Platform | Kaggle (default runtime) |
| Random Seed | 42 (used for all experiments) |

Table 6: System hardware components

| Hardware | Purpose |
| --- | --- |
| Raspberry Pi-4B | Used to run the model predictions and send alerts via SMTP email |
| Relay Module | Used to trigger an external device based on model predictions |

Table 7: Deployment Feasibility metrics

| **Metric** | **Observed Value** | **Evidence Description** |
| --- | --- | --- |
| Inference latency (per sample) | 8–15 ms | Measured during on-device TensorFlow Lite inference |
| End-to-end system latency | <200 ms | From feature input to alert trigger |
| Continuous runtime | >24 hours | Stable execution without crashes |
| CPU utilization | 22–35% | During continuous inference |
| Memory usage | ~180 MB | Including OS and runtime overhead |
